# Microtubule Actin Cross-Linking Factor 1 Regulates Cardiomyocyte Microtubule Distribution and Adaptation to Hemodynamic Overload

**DOI:** 10.1371/journal.pone.0073887

**Published:** 2013-09-26

**Authors:** John T. Fassett, Xin Xu, Dongmin Kwak, Huan Wang, Xiaoyu Liu, Xinli Hu, Robert J. Bache, Yingjie Chen

**Affiliations:** Cardiovascular Division and Lillehei Heart Institute, University of Minnesota Medical School, Minneapolis, Minnesota, United States of America; Max-Delbrück Center for Molecular Medicine (MDC), Germany

## Abstract

Aberrant cardiomyocyte microtubule growth is a feature of pressure overload induced cardiac hypertrophy believed to contribute to left ventricular (LV) dysfunction. Microtubule Actin Cross-linking Factor 1 (MACF1/Acf7) is a 600 kd spectraplakin that stabilizes and guides microtubule growth along actin filaments. MACF1 is expressed in the heart, but its impact on cardiac microtubules, and how this influences cardiac structure, function, and adaptation to hemodynamic overload is unknown. Here we used inducible cardiac-specific MACF1 knockout mice (MACF1 KO) to determine the impact of MACF1 on cardiac microtubules and adaptation to pressure overload (transverse aortic constriction (TAC).In adult mouse hearts, MACF1 expression was low under basal conditions, but increased significantly in response to TAC. While MACF1 KO had no observable effect on heart size or function under basal conditions, MACF1 KO exacerbated TAC induced LV hypertrophy, LV dilation and contractile dysfunction. Interestingly, subcellular fractionation of ventricular lysates revealed that MACF1 KO altered microtubule distribution in response to TAC, so that more tubulin was associated with the cell membrane fraction. Moreover, TAC induced microtubule redistribution into this cell membrane fraction in both WT and MACF1 KO mice correlated strikingly with the level of contractile dysfunction (r^2^ = 0.786, p<.001). MACF1 disruption also resulted in reduction of membrane caveolin 3 levels, and increased levels of membrane PKCα and β1 integrin after TAC, suggesting MACF1 function is important for spatial regulation of several physiologically relevant signaling proteins during hypertrophy. Together, these data identify for the first time, a role for MACF1 in cardiomyocyte microtubule distribution and in adaptation to hemodynamic overload.

## Introduction

Hemodynamic overload, resulting from conditions such as hypertension or aortic stenosis, elevates mechanical stress within the walls of the contracting ventricle. Increased ventricular wall stress activates signaling pathways in cardiomyocytes that promote protein synthesis and cell enlargement, leading to an increase in heart muscle mass (cardiac hypertrophy). In addition to increased protein and lipid biosynthesis, cardiomyocyte enlargement requires expansion and reorganization of the microtubule cytoskeleton [1], Composed of polymerized dimers of α- and β-tubulin, microtubules (MTs) form long, hollow cylinders that regulate cell shape and intracellular organization. An intact and extensive microtubule network is necessary for proper subcellular distribution of proteins[2], mRNAs[3] and organelles[4]. Preserving microtubule trafficking functions in the growing cardiomyocyte is therefore likely to be important for adaptation to hypertrophic stress. However, chronic hemodynamic overload causes stabilization and aberrant growth of cardiomyocyte microtubules that is believed to contribute to contractile dysfunction and the development of heart failure [5], [6], [7], [8].

The mechanism(s) by which changes in the microtubule cytoskeleton contribute to contractile dysfunction are not totally clear. One deleterious effect of excessive MT growth may be increased mechanical load due to the viscosity of the microtubule network [5], [7]. There is also recent evidence that MT interactions with T-tubules modulates sarcoplasmic reticulum ryanodine receptor sensitivity to mechanical stretch through a mechanism termed X-ROS signaling[9], [10], suggesting that excess microtubules may disrupt normal calcium dynamics in cardiomyocytes. Indeed, excess microtubules were recently implicated in disrupted calcium homeostasis in muscular dystrophy[11]. Additionally, because microtubule dependent intracellular trafficking is important for subcellular distribution of mRNAs[3], proteins[2], [12], [13] and organelles[4], changes in MT dynamics or organization could influence cardiomyocyte physiology simply by altering spatial regulation of proteins and organelles.

Microtubule-Actin Crosslinking Factor (MACF1/ACF7) is a ∼600 kd spectraplakin that guides microtubules along actin stress fibers to coordinate the cytostructural response to environmental cues[14], [15], [16]. MACF1 is expressed in numerous tissues including the skin, brain, skeletal muscle, and heart[17]. In the skin, MACF1 guidance of microtubules along stress fibers towards integrins promotes focal adhesion disassembly and allows directed cell migration[15]. Interestingly, disruption of MACF1 expression in skin had no observable effect under basal conditions, but resulted in disorganization of microtubules, enlarged focal adhesions, and impaired cell migration during wound healing[15]. In the brain, disruption of MACF1 causes defects in neurological development and perinatal death[18]. The role of MACF1 in cardiomyocyte cytoskeletal dynamics and cardiac stress handling capacity is not known. Using cardiac specific inducible disruption of MACF1 in mice, and RNAi depletion of MACF1 in isolated neonatal cardiomyocytes, we demonstrate an important role for MACF1 in cardiomyocyte microtubule organization during hypertrophy and in ventricular adaptation to hemodynamic overload.

## Materials and Methods

### Animals and transverse aortic constriction (TAC)

This study was approved by the Institutional Animal Care and Use Committee of University of Minnesota. Animals were housed in an air-conditioned room with a 12-h:12-h light-dark cycle, received standard rodent chow, and drank tap water. Floxed MACF1/ACF7 mice (MACF1^f/f^) in a c56Bl6/129 background[15] (kindly provided by Elaine Fuchs; The Rockefeller University) and crossed to Myh6-mer-cre-mer mice (Myh6-cre^+/+^) [19] (Kindly provided by Jeffrey Robbins; Cincinnati Childrens Hospital) in the c57Bl/6 background. Offspring were backcrossed to create MACF1^f/f^ /Myh6-cre^-+/^ and MACF1^f/f^ /Myh6-cre^−/−^ mice, which were used as breeders to produce litters of 50% MACF1^f/f^ /Myh6-cre^−/−^ and 50% MACF1^f/f^ /Myh6-cre^+/−^. Male littermates were treated with tamoxifen, to induce cardiomyocyte specific excision of MACF1 exons 11–13 of the MACF1/ACF7 gene (designated MACF1 KO). (Note: While the original description of these mice described the loxP sites as flanking exons 6 and 7, the most up to date NCBI map and our PCR on genomic DNA and cDNA now identify the floxed exons as 11–13, which would still result in a frameshift mutation.) MACF1^f/f^ /Myh6-cre^−/−^littermates treated with tamoxifen were used as controls (designated as WT). 4 weeks after the last tamoxifen injections, MACF1 KO and WT mice were used for sham surgery or subjected to transverse aortic constriction (TAC) as previously described [20].

### Echocardiography

Mice were anesthetized with 1.5% isoflurane and echocardiographic images were obtained with a Visual sonics high resolution Vevo 770 system as previously described [21]. LV diameter, shortening fraction and wall thickness were measured from 2-D guided short-axis M-Mode views of the LV.

### Subcellular fractionation and western blot

Subcellular fractions were isolated from mouse ventricles in glycerol based microtubule stabilization buffer as previously described[1], [5]. Briefly, ventricles were pulverized into powder under liquid nitrogen, then immediately homogenized for 15 seconds in room temperature microtubule stabilization buffer[5]. Microtubules were centrifuged at 100,000 g for 20 minutes at room temperature, and supernates were collected as the “Free fraction”, Microtubule fraction was collected by depolymerizing microtubules 2 hours at 0 degrees C in microtubule destabilization buffer[5]. After centrifugation, the supernatant was collected as the “microtubule” fraction. The remaining pellet was dissolved and resuspended by pipetting and vortexing in triton x-100 lysis buffer (20 mM Tris-HCl, 150 mM NaCl, 1 mM EDTA, 10% glycerol, 1% triton x-100, ph 7.5 + protease inhibitor cocktail (Roche) followed by centrifugation at 4 degrees C at 14000 g for 10 minutes. This step was repeated, and the combined supernatants were saved as the “membrane” fraction. The remaining pellet was resuspended in 2x SDS lysis buffer and saved as the “CSK” fraction. BCA assay was used to measure protein concentration. For comparison between different subcellular fractions, equal amounts of protein from each sample within a group were pooled to make a single representative sample of the group and run on the same gel. The cold released microtubule sample volume was based on the corresponding free fraction. Equal amounts of protein from free (cytosolic), membrane, and cytoskeletal fractions were loaded for western blot analysis.

### Western Blot

Samples were boiled 45 seconds in 1x SDS loading buffer. Longer boiling times reduced the ability to detect MACF1 by western blot. The following antibodies were used: α- tubulin, β1-integrin, src, FAK, (Cell Signaling; Beverly, MA), caveolin 3, N-cadherin, PKC α, MACF1(cat#m244), α-catenin (Santa Cruz Biotechnology; Santa Cruz, CA), α-MHC, and Desmin (Abcam; Cambridge, MA), β-MHC, β-actin (Sigma; St Louis, MO).

### RT-PCR

mRNA was isolated using Tri-reagent (Sigma; St Louis Mo). cDNA was made using random hexamers and the Advantage for RT-PCR cDNA ki (Clonetech; Mountain View, Calif). Real time PCR was performed using Fast Start Universal Syber Green Master Mix and a 7900 HT Fast Real Time PCR system (Applied Biosystems; Foster City, Calif). The following primers were used; MACF1 exon 10 forward primer: 5′ aaagaaacggaaatactggcc 3′, MACF1 exon 11 reverse primer: 5′ gcagcttaattctgccaaattc 3′. Primers used to verify exons 11–13 were deleted in MACF1 mRNA from KO hearts were the same exon 10 fwd primer as above, and a reverse primer from exon 14; 5′ aagttcagtccgggtcatcg 3′.

### Neonatal Cardiomyocyte Isolation, culture, RNAi treatment, and staining

NRVM were isolated from 2–4 day-old Sprague-Dawley rats by enzymatic digestion and separated from non-muscle cells on a discontinuous Percoll gradient as previously described [1]. Myocytes were plated on 24 well plates in serum-containing DMEM (1×10^5^ cells/cm^2^). Myocytes were incubated for between 24 and 72 hrs to allow attachment and spreading, before treatment with 10 picomoles/well MACF1 or control RNAi (Life technologies; Grand Island, NY) using RNAiMAX (Life technologies). Cells were incubated for 72 to 96 hours after RNAi treatment to allow for depletion of MACF1 protein.

For **immunofluoresent staining**, cells were fixed in methanol and stained using primary mouse tubulin antibody (sigma; St Louis, MO) followed by alexa fluor 555 conjugated secondary antibodies (Life technologies; Grand Island, NY), and hoescht stain (Life Technologies) for nuclei. Immunofluorescent staining on frozen heart sections was performed using goat anti-caveolin 3, and rabbit anti-PKCα (Santa Cruz Biotechnology), followed by alexa 555 conjugated anti-goat secondary and alexa 488 conjugated anti-rabbit secondary. Nuclei were stained using hoescht stain.

## Results

### MACF1 expression is increased in failing hearts

**Figure 1:**
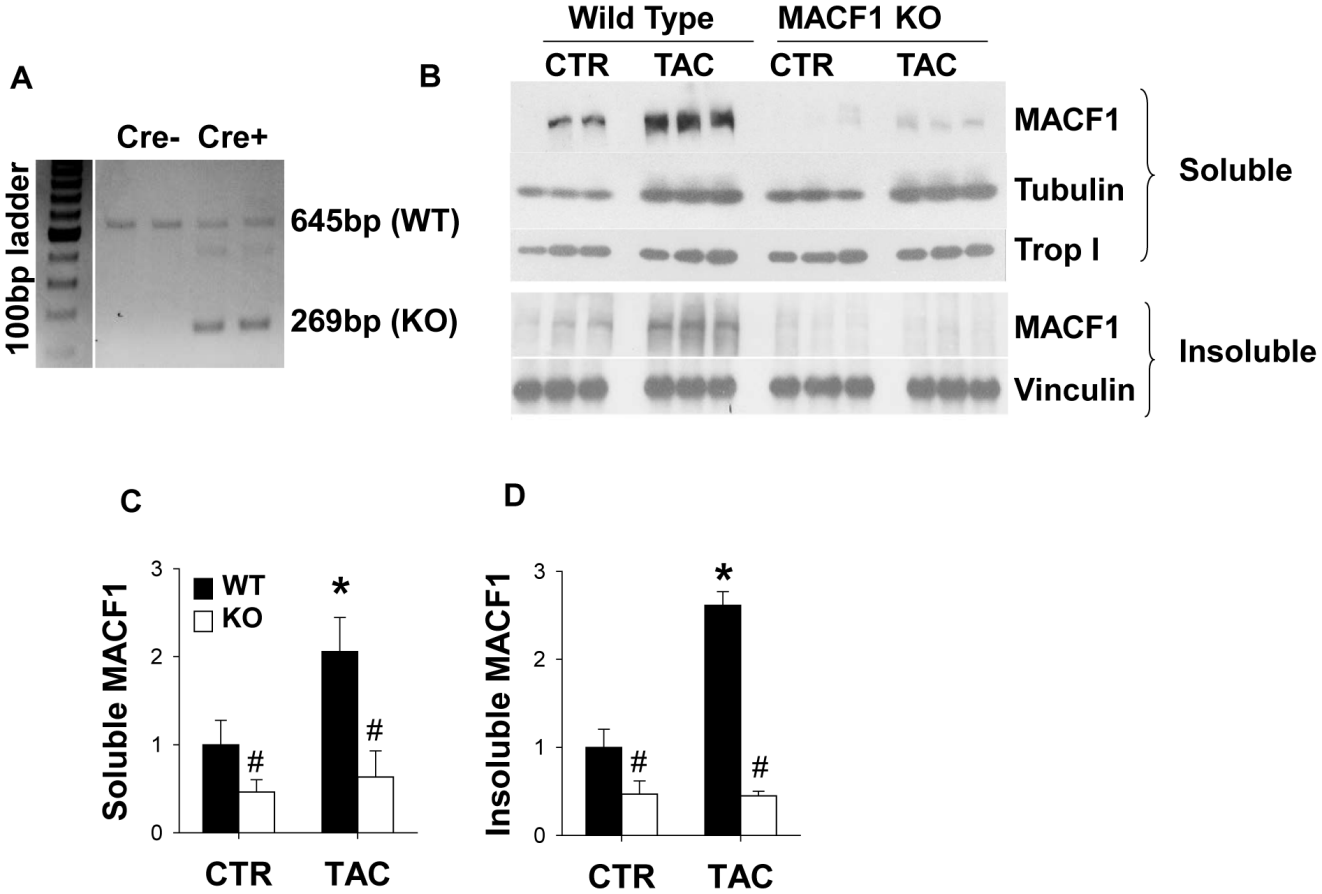
MACF1 expression is increased in response to pressure overload. RT-PCR was performed to amplify between exons 10 and 14 of MACF1. A 269 b.p. band corresponding to splicing between exon 10 and 14 (indicating loss of exons 11–13) is observed only in MACF1 KO mice, whereas WT mice express only the predicted size of 645 b.p. (Figure 1A) Western blot analysis of triton soluble (Figure 1B and 1C) and insoluble fractions (Figure 1B and 1D) of ventricular lysates of WT and MACF1 KO mice under control or after 3 weeks of TAC conditions (total of 7 weeks after tamoxifen treatment) demonstrates MACF1 expression increases in response to TAC, and is effectively silenced in MACF1 KO hearts (graphs represent averages of 3 WT or KO control mice, and 9 WT and KO TAC mice). Troponin I and vinculin were used as loading controls. (* indicates p<.05 as compared to control conditions, # indicates p<.05 as compared to WT under same conditions).

There is evidence that MACF1 is expressed in the heart[17], but its role in cardiac structure and function under basal conditions and in response to hypertrophic stress is unknown. Since global MACF1 KO is embryonic lethal[14], we used an inducible cardiomyocyte specific knockout approach to examine the role of MACF1 in cardiomyocytes. Crossing cardiac specific inducible cre (MerCreMer [19] and floxed MACF1[15] mice allows for tamoxifen inducible excision of the floxed region containing exons 11–13, resulting in a frame-shift mutation that blocks expression of MACF1[15]. PCR analysis on genomic DNA (not shown), or RT-PCR analysis of cDNA from WT and cardiac specific MACF1 KO mice demonstrated that exons 11–13 were excised in the heart in response to tamoxifen treatment (**Figure 1A**). To determine whether cardiac MACF1 expression is regulated by hypertrophic stress, we exposed WT and MACF1 KO mice to transverse aortic constriction (TAC), in which a ligature is tightened around the aorta to induce left ventricular systolic overload. Western analysis of ventricular lysates from control wild type mice or mice with TAC induced heart failure revealed that MACF1 expression was low under basal conditions, but increased nearly 2.5 fold in mice exposed to TAC (**Figure 1B, 1C and 1D**). Interestingly, quantitative RT-PCR analysis showed that MACF1 mRNA increases approximately 50% in response to TAC (not shown), suggesting MACF1 expression is regulated partially at the transcriptional level, but mostly through post-transcriptional mechanisms. Consistent with its role as both a structural microtubule associated protein (MAP) that cross-links MTs to actin, and as a MT tip binding guidance protein[22], MACF1 protein was found in both triton soluble lysates and in the triton insoluble cytoskeleton containing pellet. Expression of MACF1 was mostly abolished in MACF1 KO hearts in both soluble and insoluble fractions, suggesting the majority of MACF1 protein in the heart is expressed by cardiomyocytes. The TAC induced increase in MACF1 expression was accompanied by increased expression of tubulin, suggesting MACF1 up-regulation may play a role in the cardiomyocyte microtubule growth and remodeling process that occurs in response to pressure overload. The increase in tubulin expression was not impaired by MACF1 deletion however, and was slightly enhanced (**Figure 1B**).

### MACF1 is dispensable for normal cardiac structure and function under unstressed conditions

**Figure 2:**
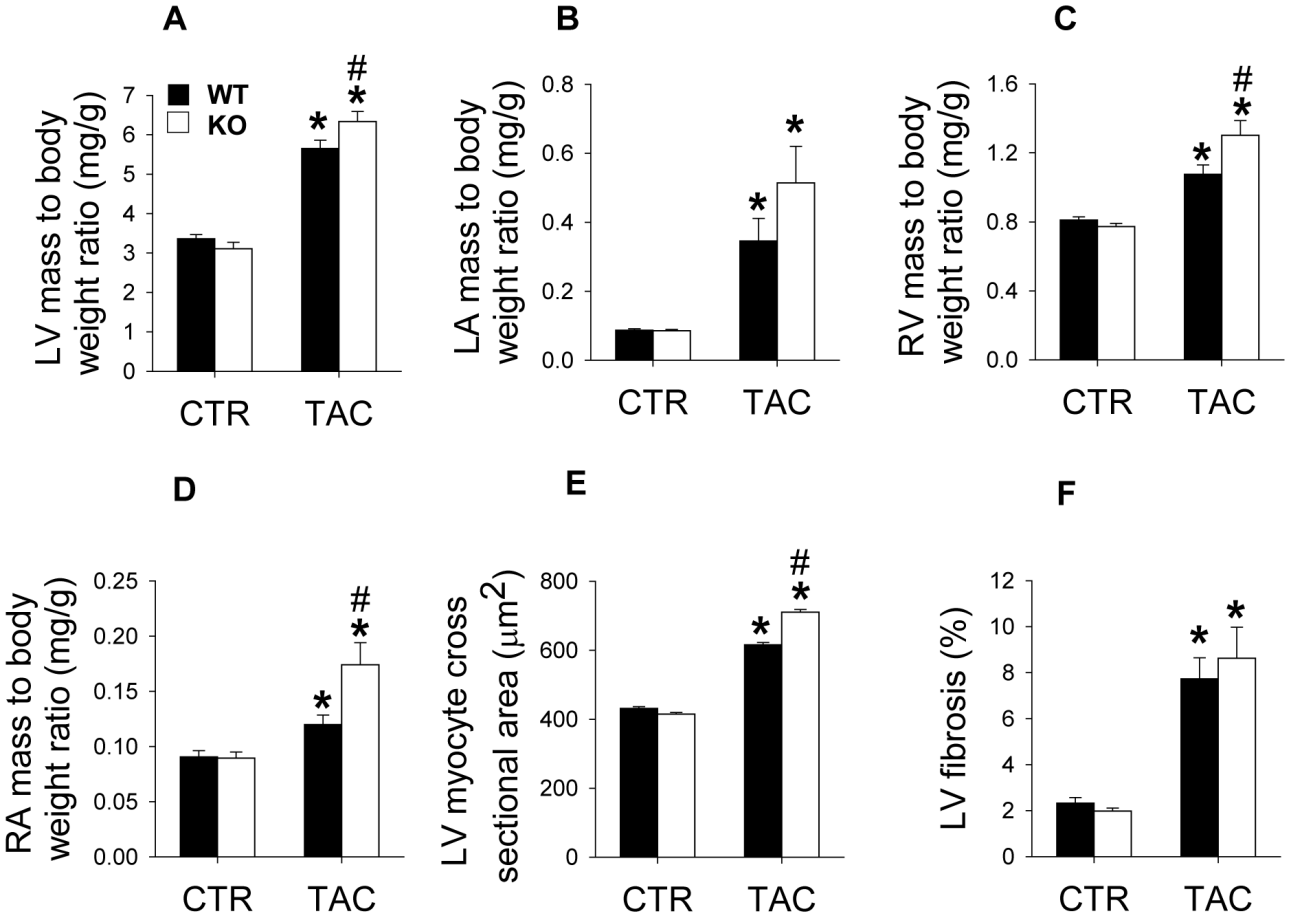
MACF1 deletion exacerbates pressure overload induced hypertrophy. 3 weeks after control or TAC conditions, WT or MACF1 KO hearts were collected and used to measure left ventricle(LV) weight to body weight ratio (Figure 2A), Left atria (LA) to body weight ratio (Figure 2B), Right ventricle (RV) weight to body weight ratio (Figure 2C) and right atria weight to body weight ratio (Figure 2D). Some hearts(n = 5 per condition) were used for measuring cardiomyocyte cross-sectional area (Figure 2E), or for Sirius red staining for fibrosis (Figure 2F) Graphs represent average tissue weights from 8 WT and 8 MACF1 KO mice under control conditions, and 19 WT and 12 MACF1 KO mice under TAC conditions. Cardiomyocyte cross sectional area was averaged from 5 mice (∼300 cells per heart) from each condition (* indicates p<.05 as compared to control conditions, # indicates p<.05 as compared to WT under same conditions).

**Figure 3:**
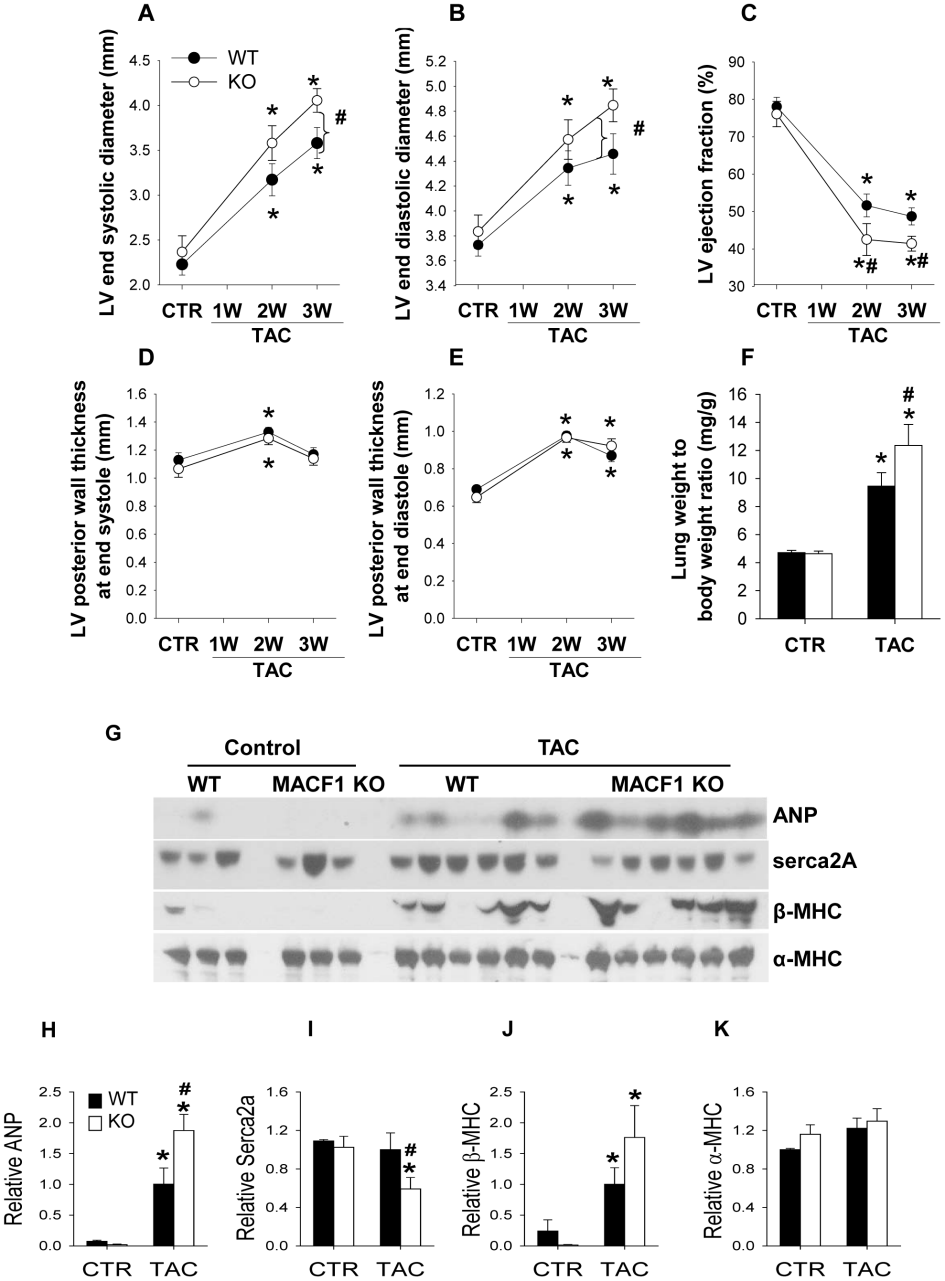
MACF1 KO exacerbates pathological features of hypertrophy in response to TAC. At 2 and 3 weeks after sham or TAC surgery, echocardiography was used to measure end systolic diameter (Figure 3A), end diastolic diameter (Figure 3B) percent ejection fraction (Figure 3C) and posterior wall thickness at end systole (Figure 3D) and end diastole (Figure 3E). After 3 weeks TAC, tissue was collected and lung weight to body weight ratio was used to indicate pulmonary congestion (Figure 3F) Expression of stress responsive proteins atrial natrurietic protein (ANP) (Figure 3G), Serca2A (Figure 3H) β-Myosin heavy chain (Figure 3I) were measured by western blot. α-MHC was used as a loading control (Figure 3J) (* indicates p<.05 as compared to control conditions, # indicates p<.05 as compared to WT under same conditions. For LV function measurements, WT control (n = 8), MACF1 KO control (n = 8), WT TAC (n = 16), KO TAC (n = 11)).

Selective MACF1 KO in adult cardiac myocytes did not cause any observable effects on LV structure, as indicated by the unchanged left ventricular weight, left atrial weight, right ventricular weight, right ventricular weight and their ratio to bodyweight (**Figures 2** **and** **3**). or tibial length (not shown) in mice. Histological staining also showed that MACF1 KO had no detectable effect on cardiac myocyte size (**Figure 2E**), and ventricular fibrosis (**Figure 2F**) under control conditions. Echocardiography can be used to measure left ventricular chamber and wall dimensions, and changes in chamber volume during contraction, allowing for analysis of ventricular function in live mice. Echocardiography measurements revealed that MACF1 KO also had no detectable effect on LV function as indicated by the normal diameters at end diastole, at end systole and LV ejection fraction in these mice (**Figure 3**).

### MACF1 KO exacerbated pressure overload-induced LV hypertrophy

While MACF1 deletion did not appear to significantly influence LV structure or function under basal conditions, the increased expression of MACF1 in response to TAC (**figure 1**) could indicate MACF1 plays a specific role in microtubule growth or reorganization that occurs during pressure overload hypertrophy. Therefore, LV structure and function were examined in WT and MACF1 KO mice subjected to severe transverse aortic constriction (TAC) for 3 weeks. Severe TAC caused ∼70% increase in LV weight, and the ratio to bodyweight (**Figure 2A**) or tibia length (not shown) in WT mice. Contrary to our expectations, MACF1 KO significantly exacerbated TAC-induced cardiac hypertrophy, as indicated by greater increase of LV weight and the ratio to bodyweight (**Figure 2A**) or tibia length (not shown) in MACF1 KO mice. MACF1 KO also significantly exacerbated TAC-induced hypertrophy of left atria, right ventricle and right atria as indicated by their weight or their ratios to bodyweight (**Figure 2B-2D**) or tibial length (not shown), indicating significantly more cardiac hypertrophy occurs in MACF1 KO hearts than in WT hearts in response to TAC. Histological tests further revealed that TAC resulted in significantly greater LV cardiomyocyte hypertrophy in MACF1 KO mice as compared to WT mice (**Figure 2E**). Myocardial fibrosis however, as evidenced by sirus red stain, was increased in response to TAC to a similar extent in WT and MACF1 KO mice (**Figure 2F**).

### MACF1 KO exacerbated TAC-induced LV chamber dilation, contractile dysfunction, and pulmonary congestion

Pathological hypertrophy is often accompanied by ventricular dilation and reduced ejection fraction, in which the ventricular chamber volume increases, but the fraction of blood pumped out of the heart is reduced. Echocardiography measurements indicated that hypertrophy in MACF1 KO mice was accompanied by significantly increased chamber dilation (**Figure 3A and 3B**) and significantly more contractile dysfunction, as indicated by reduced ejection fraction (**Figure 3C**). TAC induced changes in LV end systolic and diastolic wall thickness however, were similar in WT and MACF1 KO mice (**Figure 3D,E**). In support of an accelerated transition into congestive heart failure, MACF1 KO mice had significantly greater pulmonary congestion as indicated by lung weight to body weight ratio (**Figure 3F**). Analysis of stress markers related to heart failure in mice by western blot revealed that expression of cardiac stress marker atrial natriuretic protein (ANP) was significantly increased in MACF1 KO hearts as compared to WT hearts exposed to TAC (**Figure 3G, H**), while Serca2a protein, which is often reduced in heart failure, was significantly lower in MACF1 KO hearts after TAC than in WT hearts (**Figure 3G, I**). β-myosin heavy chain (β-MHC), which is up-regulated in pathological hypertrophy in rodents, was expressed at higher levels in MACF1 KO mice in response to TAC (**Figure 3G, J**), but this difference did not reach significance(p = 0.09). In comparison, α-myosin heavy chain expression was not significantly altered by TAC or by disruption of MACF1 (**Figure 3G, K**). Together, the results in **figures 2** and 3 indicate that MACF1 disruption exacerbates pathological hypertrophy in response to pressure overload.

### Maladaptive response to pressure overload is associated with redistribution of microtubules into distinct subcellular fractions

**Figure 4:**
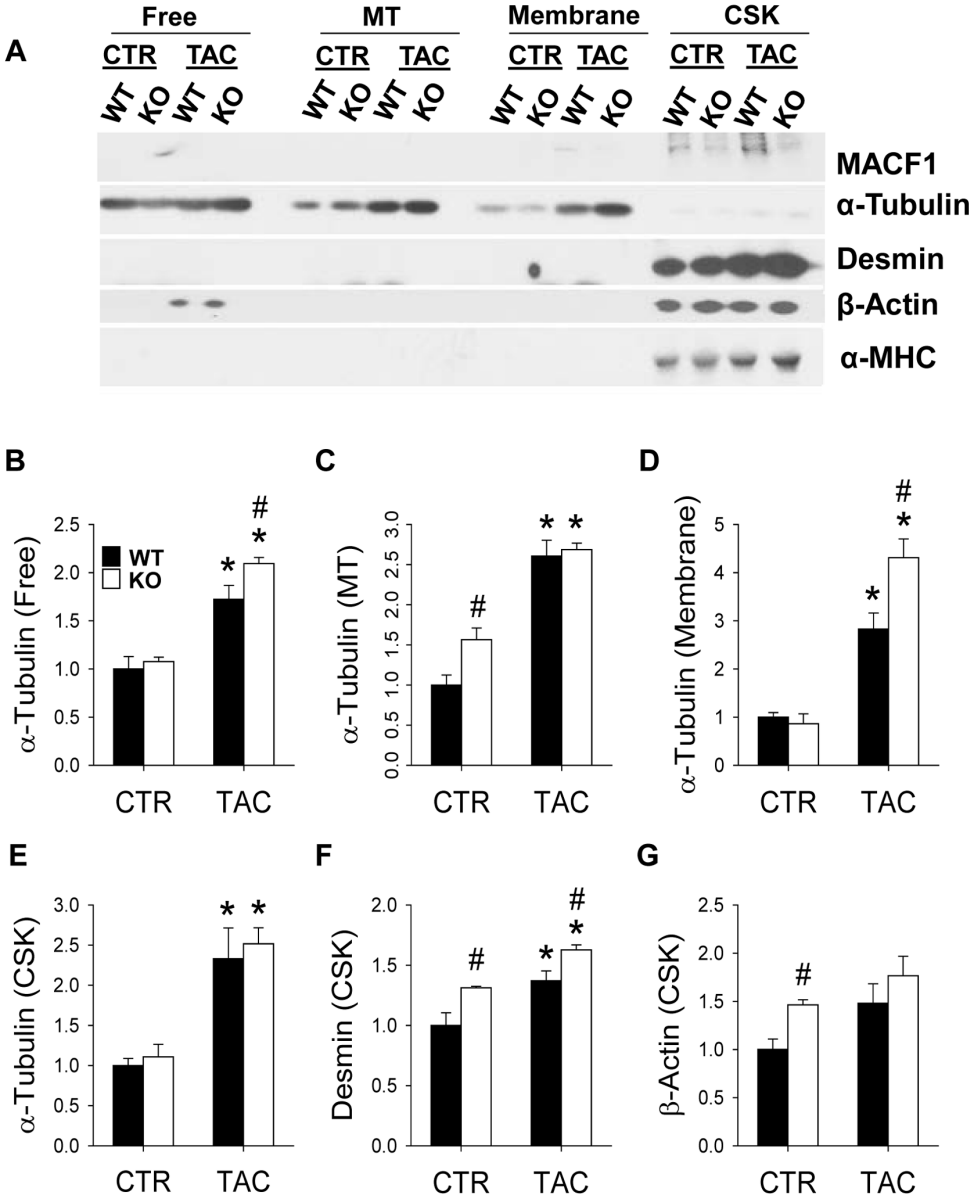
Maladaptive response to pressure overload is associated with redistribution of microtubules into distinct subcellular fractions. LV lysates from WT and MACF1 KO hearts exposed to control or TAC conditions were separated into cytosolic fractions (Free), Microtubule fractions (MT), trition soluble membrane fractions (Membrane), and triton insoluble cytoskeletal fraction(CSK) Equal amounts of fractionated proteins from each sample in a treatment group were pooled and the four different fractions were examined side by side by western blot to measure relative changes in levels and subcellular distribution of MACF1, Tubulin, Desmin and β-Actin (Figure 4A–4G) (* indicates p<.05 as compared to control conditions, # indicates p<.05 as compared to WT under same conditions).

The results in figures 2 and 3 suggest MACF1 expression is important for physiological adaptation to pressure overload. To determine if the detrimental effects of MACF1 deletion were related to its effects on cardiac microtubules, we examined microtubule levels and distribution as well as other extra-sarcomeric cytoskeletal elements in WT and MACF1 KO hearts. Ventricular lysates of WT and MACF1 KO mice were separated into free cytosolic fractions (Free), microtubule enriched fractions (MTs), triton soluble membrane fractions (Membrane), and triton insoluble cytoskeletal fractions (CSK) as previously described[1], so that changes in expression and distribution of cytoskeletal elements, as well as other hypertrophy related proteins could be analyzed (**Figure 4**).

**Figure 5:**
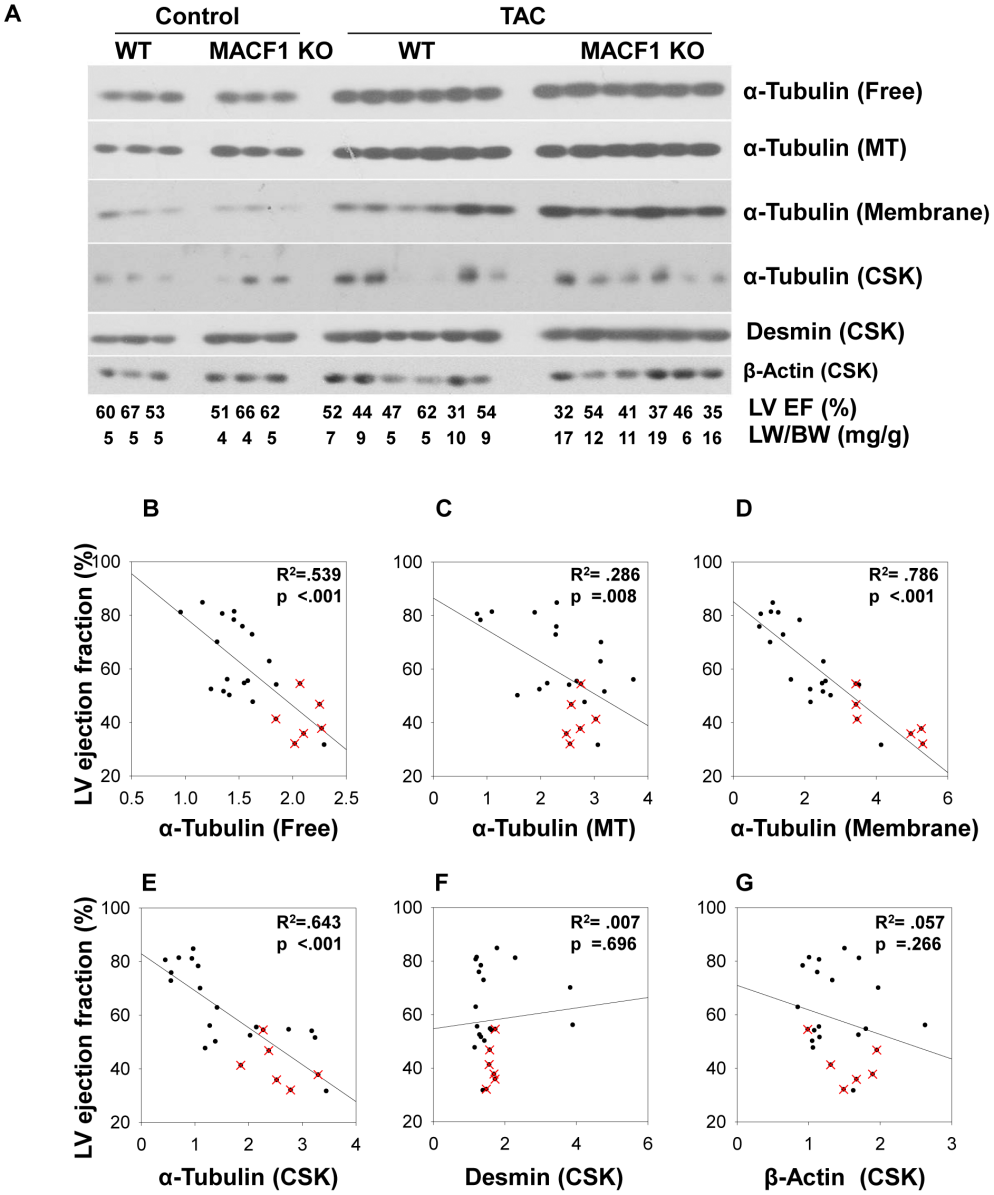
Maladaptive response to pressure overload is associated with redistribution of microtubules into distinct subcellular fractions. The relationship between LV ejection fraction and the levels of Tubulin, as well as β-actin, and Desmin, in different subcellular fractions (Figure 5A) was examined by linear regression analysis (Figure 5B–5G). The data was collected from 18 WT mice and 6 MACF1 KO mice exposed to TAC. Relative values of the protein levels were determined as fold increase over non-banded controls from the same experiment. Samples with a red x are MACF1 KO.

Interestingly, when different subcellular fractions were loaded equally and compared side by side, MACF1 appeared mostly associated with the cytoskeletal fraction, while little tubulin appears in this fraction, suggesting MACF1 may be persistently linked to the actin cytoskeleton, intermediate filaments, or other triton insoluble elements in cardiomyocytes (**Figure 4A**). Surprisingly, despite the low level of MACF1 expression under basal conditions, higher levels of polymerized microtubules, and slightly increased desmin and β-actin were observed in MACF1 KO hearts as compared to WT hearts (**Figure 4A, B,G, and H** **,** **Figure 5**). This suggests that a low level of cytoskeletal remodeling occurred in response to MACF1 gene disruption, but these changes were not sufficient to alter heart size or function under basal conditions. In response to TAC, levels of free tubulin increased by 70% in WT hearts, and ∼100% in MACF1 KO hearts, while cold-labile MTs increased similarly in both WT and MACF1 KO hearts (∼160% increase) (**Figure 4A–C** **,** **Figure 5**). Cytoskeletal desmin also increased in response to TAC similarly in WT and MACF1 KO hearts (∼30% increase) so that the slightly higher desmin levels in MACF1 hearts under basal conditions was maintained after TAC (**Figure 4A, B and G** **,** **Figure 5**). Interestingly, the largest TAC induced difference observed in the cytoskeletal proteins examined was the increase in tubulin associated with the membrane fraction (∼180% increase in WT TAC compared to control) (**Figure 4A, B and E** **,** **Figure 5**). Redistribution of tubulin into the membrane fraction was further elevated by MACF1 KO (∼330% increase over control in MACF1 KO), suggesting a specific effect of MACF1 on microtubule distribution.

In support of a potential relationship between TAC induced contractile impairment and microtubule redistribution, we observed that hearts collected from mice with lower LV ejection fractions tended to have higher levels of membrane associated tubulin (**Figure 5A**). Linear regression analysis of the relationship between tubulin levels in different subcellular fractions and LV ejection fraction (**Figure 5B–5G**) in 6 WT and 6 MACF1 KO mice from this study, as well as 12 additional WT mice from two previous studies[1], [23] (File S1 & S2), revealed a strong and significant correlation between increased membrane associated tubulin and reduced ejection fraction (r^2^ = 0.786, p<.001) (**Figure 5A and 5D**). Lung weight to body weight ratio, an indicator of pulmonary congestion resulting from LV dysfunction, also correlated positively with membrane associated tubulin (r^2^ = 0.686, p<.001). As we have previously observed [1], the levels of triton insoluble tubulin (CSK) in WT mice also showed a strong correlation with reduced ejection fraction (r^2^ = 0.643, p<.001) (**Figure 5E**) but this relationship was slightly diminished in MACF1 KO mice. Cold released tubulin (MTs) did not demonstrate as strong a correlation with LV dysfunction (**Figure 5C**), suggesting microtubule redistribution, rather than microtubules per se, may play a greater role in pressure overload LV dysfunction. Together these results demonstrate that MACF1 regulates microtubule distribution in cardiomyocytes, and identify a novel relationship between microtubule distribution into cell membrane fractions and LV dysfunction.

### MACF1 regulates β1 integrin, caveolin 3, and PKCα content in the cell membrane

**Figure 6:**
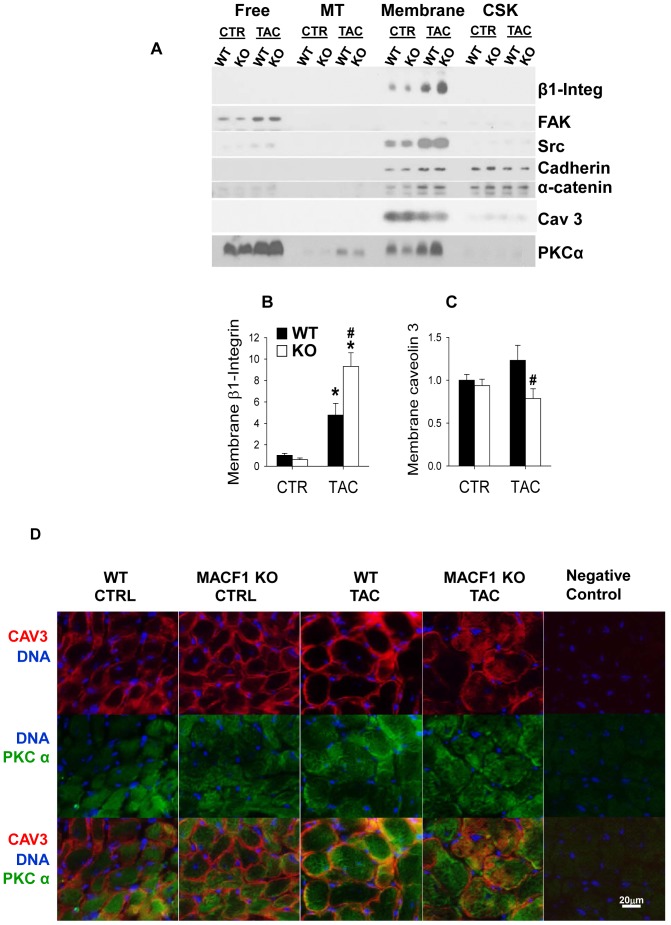
MACF1 KO alters distribution of β1 integrin, caveolin 3, and PKCα content in the cell membrane fraction. Western analysis of focal adhesion proteins β1 integrin, FAK and SRC, cell/cell adhesion proteins N-cadherin, and α-catenin, as well as caveolin-3 and PKCα content from pooled subcellular fractions of WT and MACF1 KO hearts (Figure 6A). Levels of each protein were also examined in individual samples within a fraction to determine if observed differences were statistically significant. β1 integrin (Figure 6B) and caveolin 3 (Figure 6C) were mostly localized to the cell membrane. PKCα protein was most abundant in the cytosol, but was significantly elevated in the membrane fraction of MACf1 KO mice exposed to TAC. Immunofluorescence analysis of PKCα (green) and caveolin 3 (red) in heart cross-sections (Figure 6D) revealed that caveolin 3 membrane distribution is disrupted in MACF1 KO hearts after TAC. Hoescht stain (blue) identifies nucleii (* indicates p<.05 as compared to control conditions, # indicates p<.05 as compared to WT under same conditions, n = 4 to 6 per condition; n = 4–6 per group.)

MACF1 has been demonstrated to direct microtubules along actin filaments towards integrins to disrupt focal adhesions to allow for cell migration[15]. Additionally, microtubule interaction with focal adhesions was shown to be important for delivery of caveolin to the cell membrane [24]. We hypothesized that MACF1 disruption might impair these processes, and that continued signaling from focal adhesions might contribute to the increased hypertrophy observed in MACF1 KO hearts. Western blot analysis of subcellular fractions of ventricular lysates revealed that TAC increased the levels of β1 integrin, FAK, and SRC in both WT and MACF1 KO hearts. (**Figure 6A**) Interestingly, MACF1 KO further increased β1 integrin levels in membrane fractions (**Figure 6A and 6B**), but had no observable effect on FAK and SRC levels or distribution as compared to WT mice. Analysis of triton x-100 lysates to examine phospho-signaling related to focal adhesions also revealed no difference in FAK^Tyr397^ or SRC^Tyr416^ phosphorylation (data not shown), suggesting that even though β1 integrin levels were elevated, this did not result in greater activation of focal adhesion signaling.

In contrast to β1 integrin, caveolin 3 was significantly reduced in cell membrane fractions of MACF1 KO hearts after TAC. (**Figure 6A and 6C**). These results are consistent with a role for MACF1 guidance of MTs to focal adhesions, not only for disruption of focal adhesions, but also for delivery of caveolin-3 to the cell membrane. To further characterize how MACF1 altered distribution of caveolin-3 and PKCα in the heart, we examined caveolin-3 and PKCα distribution in frozen heart cross-sections from WT and MACF1 KO mice under basal or TAC conditions (**Figure 6D**). Immunofluorescent analysis did not reveal obvious differences in PKCα distribution in WT or MACF1 KO hearts under basal or TAC conditions, possibly because cytoplasmic expression is so high. Immunofluorescent analysis of caveolin-3 revealed that caveolin-3 distribution was mostly localized to the sarcolemma in WT and MACF1 KO hearts under basal conditions. In WT mice exposed to TAC, caveolin-3 localization to the sarcolemma was maintained. In MACF1 KO hearts however, sarcolemmal caveolin-3 distribution was disrupted in response to TAC, so that more caveolin-3 localized in the cytoplasm and was more reduced at the sarcolemma. Because caveolin-3 levels are reduced in MACF1 KO hearts, but still very abundant in the cell membrane fraction, it is possible that the caveolin-3 observed in the cytoplasm is associated with membrane bound vesicles that were not appropriately trafficked to the sarcolemma.

**Figure 7:**
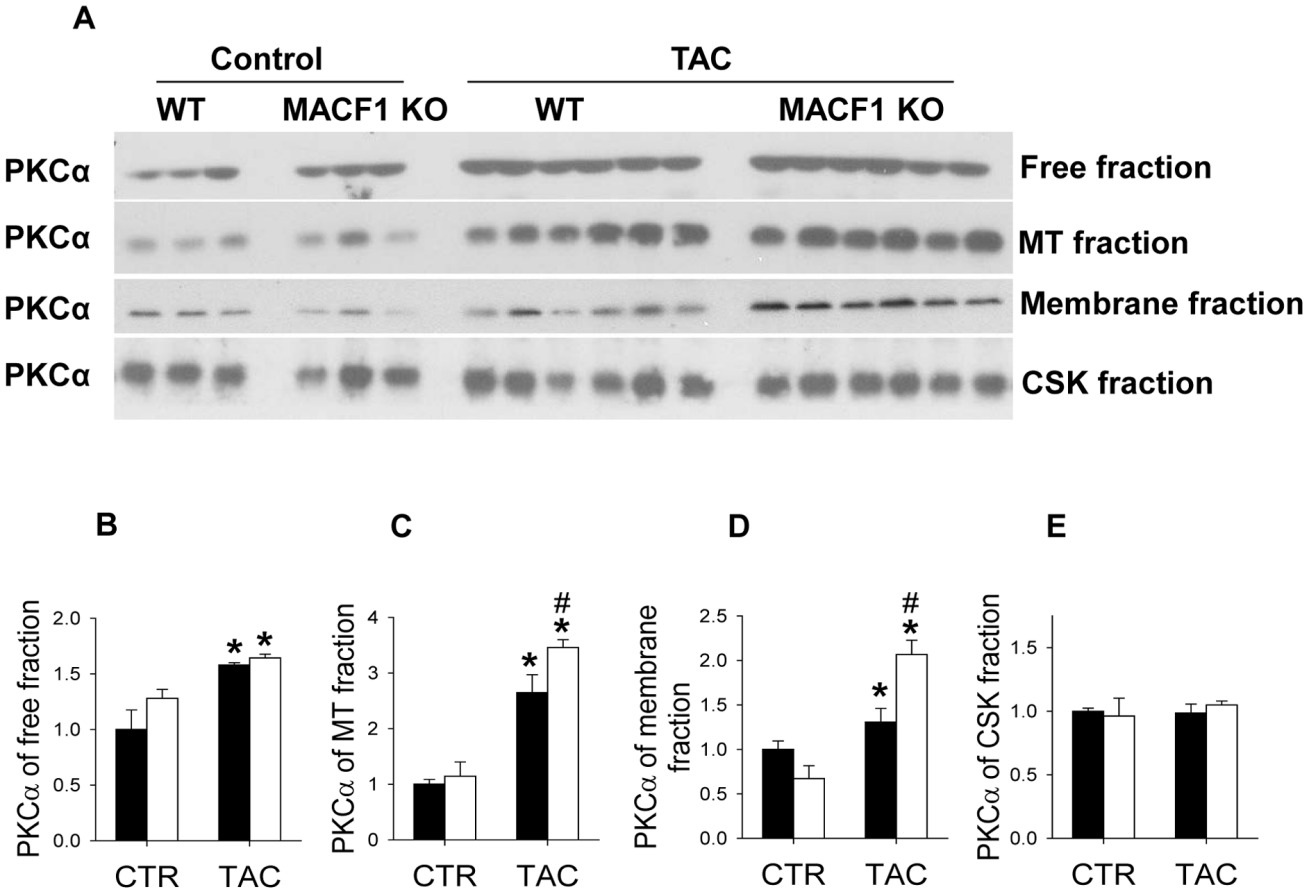
MACF1 KO increases membrane abundance of PKCα. Western blot analysis of PKCα in subcellular fractions of ventricular lysates from WT and MACF1 KO hearts (**Figure 7A–E**). (* indicates p<.05 as compared to control conditions, # indicates p<.05 as compared to WT under same conditions, n = 4 to 6 per condition; n = 4–6 per group.)

PKCα is another protein that is regulated by the microtubule cytoskeleton [25], [26] that is believed to play a role in pathological hypertrophy [27]. PKCα expression was most abundant in the Free fraction, but also was observed at moderate levels in MT and membrane associated fractions (**Figure 6A**), and increased significantly in response to TAC in both WT and MACF1 KO hearts. PKCα associates with cell membranes upon activation, and there is some evidence that its degradation is microtubule and caveolae dependent [28], [29]. Interestingly, while levels of free PKCα increased similarly in WT and MACF1 KO mice in response to TAC, membrane associated PKCα was significantly elevated in MACF1 KO hearts (**Figure 7A, B, and D**).

Together, these results suggest MACF1 regulation of microtubule organization oppositely influences levels and distribution of PKCα and caveolin 3 under stress conditions, which may help account for the increased hypertrophy and worse dysfunction observed in MACF1 KO hearts.

### MACF1 is necessary for neonatal cardiomyocyte microtubule organization

**Figure 8:**
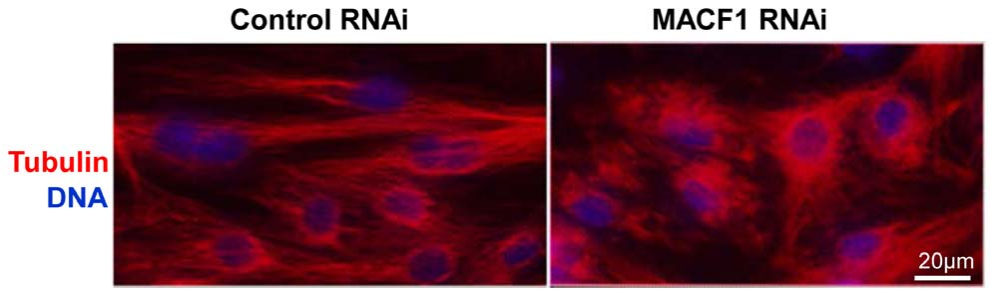
MACF1 depletion alters microtubule organization in neonatal rat cardiomyocytes. Neonatal rat ventricular cardiomyocytes(NRVMs) transfected with MACF1 RNAi or non-targeting control RNAi were immunostained for tubulin (red). Hoescht stain (blue) was used to identify nuclei.

To better understand how MACF1 disruption might be altering microtubule distribution, and subsequent changes in subcellular organization, we used RNAi to knock down MACF1 in neonatal rat cardiomyocytes. Interestingly, MACF1 RNAi added one day after plating severely disrupted microtubule organization, prevented MT extension, and dramatically limited cell spreading over the next 72 hours as compared to cells transfected with a non-targeting control RNA (not shown). Because early MACF1 knockdown after plating prevented cell spreading, we allowed cardiomyocytes to spread for 3 days in 10% FCS prior to treating with RNAi, so that changes in microtubule organization between MACF1 and control RNAi treatment would be comparable independent of dramatic differences in cell shape. Under these conditions, MACF1 RNAi treatment still disrupted cardiomyocyte MT organization, so that microtubules appeared curled and tangled as compared to the longer, semi-parallel microtubules observed in control cells (**Figure 8**). The disruption of normal microtubule extension and organization by MACF1 depletion we observe in vitro may help explain how microtubule dependent distribution and regulation of sarcolemmal proteins such as caveolin 3 is disrupted in vivo.

## Discussion

MACF1 plays an important role in microtubule organization in epidermal cells[15], skin stem cells[30], and neurons[18], [22]. Here we demonstrate for the first time that MACF1 is up-regulated in the heart in response to pressure overload. While MACF1 expression in cardiac myocytes is dispensable for normal heart structure and function in mice under basal conditions, MACF1 KO significantly exacerbated TAC induced LV hypertrophy, LV dilation and contractile dysfunction. The increase in microtubule redistribution into triton soluble membrane fractions in MACF1 KO hearts in response to stress, and strong correlation with LV dysfunction, suggests MACF1 regulation of microtubule organization is important for ventricular adaptation to hemodynamic overload.

MACF1 disruption in adult heart caused no observable differences in heart size or function. We did observe slight increases in desmin, β-actin and polymerized microtubules under basal conditions in MACF1 KO hearts, suggesting disruption of MACF1 did affect cytoskeletal remodeling in adult cardiac myocytes in vivo, though these changes are insufficient to alter cardiomyocyte function. The low level of MACF1 expression in the adult heart under basal conditions and significant increase in expression in response to pressure overload, along with up-regulated tubulin and prior demonstration of increased MAP4 expression[31], suggests MACF1 up-regulation is part of a concerted microtubule remodeling response of cardiomyocytes under hypertrophic stress.

The finding that MACF1 KO exacerbated hypertrophy and LV dysfunction in response to pressure overload suggests MACF1, and presumably it’s influence on microtubule organization, plays an important role in cardiomyocyte adaptation to stress. While microtubule levels and free tubulin increased significantly in both WT and MACF1 KO hearts in response to TAC, there was little difference between WT and MACF1 KO hearts in these measurements, suggesting microtubule levels per se were not the cause of worse dysfunction in MACF1 KO hearts. The greatest change observed in extra-sarcomeric cytoskeletal protein expression or distribution was the increase in tubulin associated with the triton soluble membrane fraction. Membrane associated tubulin was increased 180% in response to TAC in WT hearts, while MACF1 KO hearts exhibited a 330% increase in membrane associated tubulin(**Figure 4**). Furthermore, we observed that the increase in tubulin found in these fractions correlated strikingly with reduced ejection fraction (**Figure 5**). We also observed a strong correlation between tubulin associated with the triton insoluble fraction and LV dysfunction, and the levels of tubulin in this fraction were not significantly reduced by MACF1 KO. While these correlations do not demonstrate whether tubulin redistribution is a cause or consequence of LV dysfunction, our previous findings using colchicine did demonstrate that reducing MT polymerization attenuates development of TAC induced hypertrophy and heart failure[1]. Together these results demonstrate that MT redistribution to cell membranes, rather than increased microtubules per se, is most closely associated with pressure overload induced contractile dysfunction, and that MACF1 expression is important for limiting aberrant MT-membrane interactions. Further experiments will be necessary however, to determine the specific mechanism(s) by which excess microtubule growth and or altered distribution contributes to LV dysfunction.

Based upon the previously described role of MACF1 in guiding MTs toward focal adhesions[15], we hypothesize that the increased membrane associated tubulin in MACF1 KO hearts results from misguided growth of microtubules that form aberrant interactions with membrane bound organelles, and that these interactions occur less frequently when MACF1 is present to guide microtubule growth along actin filaments. While we were unable to stain microtubules in frozen sections with sufficient resolution to distinguish how MACF1 KO altered MT distribution in the heart, we were able to observe the effects of RNAi mediated MACF1 depletion on microtubule organization in cultured neonatal rat cardiomyocytes. Compared to control cells, which formed microtubules that extended to the cell periphery, microtubules in MACF1 depleted cells were highly disorganized, and displayed crimped or crooked trajectories that resulted in perinuclear MT densification (**Figure 8**). The reduced ability of microtubules to extend in absence of MACF1 suggests the increased abundance of membrane associated tubulin observed in the heart in vivo may be the result of aberrant MT interactions with intracellular membranes, and that MACF1 guidance of MTs along actin filaments helps preserve normal MT distribution. Aberrant distribution of microtubules is likely to alter distribution of proteins that depend on organized microtubule trafficking. Additionally, microtubule interaction with the sarcolemma and T-tubules has recently been implicated in mechanical regulation of calcium handling in muscle cells through a mechanism termed X-ROS signaling [10]. Dysregulation of this pathway appears to result from abnormal microtubule accumulation or organization, and has recently been implicated as an underlying factor in the pathology of duchenne muscular dystrophy [11]. Further experiments to determine which cardiomyocyte membranes or membrane proteins tubulin is associating with in response to pressure overload, and whether this might be related to X-ROS signaling are underway.

Because microtubules play an important role in subcellular distribution of proteins and organelles, we also examined expression and localization of hypertrophy related proteins that are regulated by microtubule trafficking and which might be altered by MACF1 disruption. Interestingly, MACF1 KO reduced membrane caveolin 3, but increased membrane PKCα and β1 integrin as compared to WT mice after TAC. These changes in response to MACF1 disruption are consistent with a disorganized microtubule network in which trafficking of caveolin 3 to focal adhesions is impaired, and endosomal degradation of active membrane PKCα is decreased. Because PKCα contributes to cardiac hypertrophy and LV dysfunction in pressure overload [27], while loss of caveolin 3 promotes cardiomyopathy [32], the altered expression and distribution of these proteins may help explain the increased pathological hypertrophy observed in MACF1 KO mice after TAC.

In conclusion, our data identify MACF1 as a stress induced regulator of cardiomyocyte microtubule distribution that is important for ventricular adaptation to hemodynamic overload.

## Supporting Information

File S1 **Previous manuscripts from which some parts of the data in** **figure 5** **were obtained.**
(PDF)

File S2 **Previous manuscripts from which some parts of the data in** **figure 5** **were obtained.**
(PDF)
