# Supplementary material for: Microtubule Actin Cross-Linking Factor 1 Regulates Cardiomyocyte Microtubule Distribution and Adaptation to Hemodynamic Overload
Source: PLoS One. 2013 Sep 26;8(9):e73887. doi: 10.1371/journal.pone.0073887 (PMC3784444; doi:10.1371/journal.pone.0073887)
Supplement: File S1 — Previous manuscripts from which some parts of the data in figure 5 were obtained. (PDF) [file pone.0073887.s001.pdf]

Adenosine regulation of microtubule dynamics in cardiac hypertrophy

John T. Fassett, Xin Xu, Xinli Hu, Guangshuo Zhu, Joel French, Yingjie Chen and Robert J. Bache

Am J Physiol Heart Circ Physiol 297:H523-H532, 2009. First published 12 June 2009;
doi: 10.1152/ajpheart.00462.2009

You might find this additional info useful...

Supplementary material for this article can be found at:
http://ajpheart.physiology.org/http://ajpheart.physiology.org/content/suppl/2009/06/29/00462.2009.DC1.html

This article cites 60 articles, 43 of which you can access for free at:
http://ajpheart.physiology.org/content/297/2/H523.full#ref-list-1

This article has been cited by 5 other HighWire-hosted articles:
http://ajpheart.physiology.org/content/297/2/H523#cited-by

Updated information and services including high resolution figures, can be found at:
http://ajpheart.physiology.org/content/297/2/H523.full

Additional material and information about ***American Journal of Physiology - Heart and Circulatory Physiology*** can be found at:
http://www.the-aps.org/publications/ajpheart

This information is current as of July 5, 2013.

American Journal of Physiology - Heart and Circulatory Physiology publishes original investigations on the physiology of the heart, blood vessels, and lymphatics, including experimental and theoretical studies of cardiovascular function at all levels of organization ranging from the intact animal to the cellular, subcellular, and molecular levels. It is published 12 times a year (monthly) by the American Physiological Society, 9650 Rockville Pike, Bethesda MD 20814-3991. Copyright © 2009 the American Physiological Society. ISSN: 0363-6135, ESSN: 1522-1539. Visit our website at http://www.the-aps.org/.

Downloaded from http://ajpheart.physiology.org/ at Univ of Minnesota on July 5, 2013

Am J Physiol Heart Circ Physiol 297: H523–H532, 2009.
First published June 12, 2009; doi:10.1152/ajpheart.00462.2009.

Adenosine regulation of microtubule dynamics in cardiac hypertrophy

John T. Fassett, Xin Xu, Xinli Hu, Guangshuo Zhu, Joel French, Yingjie Chen, and Robert J. Bache
Cardiovascular Division and Lillehei Heart Institute, University of Minnesota Medical School, Minneapolis, Minnesota

Submitted 19 May 2009; accepted in final form 12 June 2009

Fassett JT, Xu X, Hu X, Zhu G, French J, Chen Y, Bache RJ. Adenosine regulation of microtubule dynamics in cardiac hypertrophy. *Am J Physiol Heart Circ Physiol* 297: H523–H532, 2009. First published June 12, 2009; doi:10.1152/ajpheart.00462.2009.—There is evidence that endogenous extracellular adenosine reduces cardiac hypertrophy and heart failure in mice subjected to chronic pressure overload, but the mechanism by which adenosine exerts these protective effects is unknown. Here, we identified a novel role for adenosine in regulation of the cardiac microtubule cytoskeleton that may contribute to its beneficial effects in the overloaded heart. In neonatal cardiomyocytes, phenylephrine promoted hypertrophy and reorganization of the cytoskeleton, which included accumulation of sarcomeric proteins, microtubules, and desmin. Treatment with adenosine or the stable adenosine analog 2-chloroadenosine, which decreased hypertrophy, specifically reduced accumulation of microtubules. In hypertrophied cardiomyocytes, 2-chloroadenosine or adenosine treatment preferentially targeted stabilized microtubules (containing detyrosinated α-tubulin). Consistent with a role for endogenous adenosine in reducing microtubule stability, levels of detyrosinated microtubules were elevated in hearts of CD73 knockout mice (deficient in extracellular adenosine production) compared with wild-type mice (195%, $P < 0.05$). In response to aortic banding, microtubules increased in hearts of wild-type mice; this increase was exaggerated in CD73 knockout mice, with significantly greater amounts of tubulin partitioning into the cold-stable Triton-insoluble fractions. The levels of this stable cytoskeletal fraction of tubulin correlated strongly with the degree of heart failure. In agreement with a role for microtubule stabilization in promoting cardiac dysfunction, colchicine treatment of aortic-banded mice reduced hypertrophy and improved cardiac function compared with saline-treated controls. These results indicate that microtubules contribute to cardiac dysfunction and identify, for the first time, a role for adenosine in regulating cardiomyocyte microtubule dynamics.

CD73; ventricular hypertrophy; heart failure

ADENOSINE IS AN AUTACOID RELEASED in response to ischemia or other stresses that increase the catabolism of ATP. Besides its role in preconditioning and protection against ischemia-reperfusion injury (13, 30, 36, 40), adenosine protects mice exposed to chronic pressure overload secondary to transverse aortic constriction (TAC) against the development of pathological hypertrophy and heart failure (31, 58). Adenosine or adenosine receptor agonists can also inhibit hypertrophy of isolated cardiomyocytes (16, 31, 58), suggesting a direct antihypertrophic effect.

Although compensatory and/or physiological hypertrophy can occur with normal or even increased contractile function, pathologically hypertrophied hearts often progress to a decompensated state, in which contractility is depressed and heart failure ensues. There is evidence in animal models that myocardial adenosine levels increase during compensatory hypertrophy but are diminished as hearts decompensate (15, 35), raising the possibility that reduced adenosine levels may play a role in the transition from compensated hypertrophy to heart failure. In agreement with a role for endogenous adenosine in preserving contractile function, we recently observed that deletion of CD73 (the ectonucleotidase that produces extracellular adenosine from AMP) exacerbated hypertrophy and contractile dysfunction in mice subjected to TAC. However, the mechanism(s) by which adenosine could attenuate hypertrophy and preserve cardiac function is unclear.

Although numerous signaling pathways contribute to cardiomyocyte hypertrophy, increased cell size is ultimately dependent on expansion of the cytoskeletal networks that comprise the cell infrastructure. A critical structural support element of the growing cell is the microtubule cytoskeleton. Microtubules are stiff polymers of α- and β-tubulins, which radiate from origins near the nucleus and extend toward the cell periphery, sometimes anchoring to membrane proteins (3). Microtubules are critically involved in many basic cellular functions, including cell division (5), migration (43), and transport of mRNA (32), proteins (39), and organelles (42). According to the tensegrity model of cytoskeletal architecture, microtubules serve as compression-resistant elements that balance or resist contractile tension within the framework of the remaining cytoskeleton (7, 25, 26, 56). Although the majority of microtubules are dynamic, shortening or lengthening in response to external stimuli, a portion of the microtubule population may be stabilized to promote directional cell migration (38) or polarized cell growth (47). Interestingly, microtubule stabilization also occurs in response to pressure overload (44). Although an appropriate population of microtubules is essential to maintain cell shape and intracellular transport functions, an overabundance of microtubules can interfere with the contractile function of cardiomyocytes. There is evidence that accumulation of a highly dense, stable population of microtubules in cardiomyocytes is associated with a decompensated response to sustained elevation of systolic wall stress (11, 29, 49, 55). Moreover, cardiomyocyte elongation in pressure-overload-induced heart failure is associated with increased microtubule densification (57).

Here we examined the effects of adenosine on the cardiomyocyte cytoskeleton and found that extracellular adenosine specifically regulates cardiomyocyte microtubule stability during pressure overload in vivo and in response to hypertrophic stimuli in neonatal rat ventricular myocytes (NRVMs). This novel role in regulation of cardiomyocyte microtubule dynamics may help explain the protective effects of adenosine against hypertrophy and heart failure in hearts exposed to chronic pressure overload.

METHODS

Animals and TAC. Animals were housed in an air-conditioned room with a 12:12-h light-dark cycle, received standard rodent chow,

Address for reprint requests and other correspondence: R. J. Bache, Division of Cardiology, Univ. of Minnesota, Mayo Mail Code 508, 420 Delaware St. SE, Minneapolis, MN 55455 (e-mail: bache001@umn.edu).

 0363-6135/09 $8.00 Copyright © 2009 the American Physiological Society

Downloaded from http://ajpheart.physiology.org/ at Univ of Minnesota on July 5, 2013

and drank tap water. CD73 gene-deficient [$CD^{-/-}$ (CD KO)] mice (129 background) and control wild-type (WT) mice were generated and used for the sham procedure or subjected to TAC, as previously described (24). This study was approved by the Institutional Animal Care and Use Committee of the University of Minnesota.

Colchicine treatment of Balb/c mice. Colchicine was injected intraperitoneally 3 days after the TAC procedure; the injections began with 0.4 mg/kg and progressed to 1 mg/kg to allow the mice to adjust to the drug, similar to a previously described protocol (54), as follows: 0.4 mg/kg on *day 3*, 0.6 mg/kg on *day 5*, 0.8 mg/kg on *day 7*, 1 mg/kg on *day 10*, and 1 mg/kg on *day 12*. Tissue was collected on *day 14*. Control aortic-banded mice were injected with saline.

Echocardiography. Mice were anesthetized with 1.5% isoflurane, and echocardiographic images were obtained with a Visual Sonics high-resolution Veve 660 system, as previously described (60). Left ventricular (LV) diameter, shortening fraction, and wall thickness were measured from two-dimensional guided short-axis M-mode views of the LV.

NRVM isolation and culture. NRVMs were isolated from 2-day-old Sprague-Dawley rats by enzymatic digestion (61) and separated from nonmuscle cells on a discontinuous Percoll gradient according a modified protocol from Dr. U. Mende (Cardiovascular Division, Brigham and Women's Hospital) (61; also see supplemental information in the online version of this article.)

Isolation of cytoskeletal fractions from NRVMs and Western blot. Triton-soluble fractions (containing cytosolic and membrane proteins) were gently extracted from the adherent cells in 1% Triton X-100 lysis buffer; the remaining Triton-insoluble fraction (containing intact cytoskeleton) was collected in 2× SDS loading buffer (see supplemental information).

Isolation of microtubules and other fractions from mouse cardiac tissue. Mouse hearts were processed for separation of free and polymerized tubulin, as previously described (55) with minor modifications (see supplemental information).

Chemicals and antibodies. Phenylephrine (PE), adenosine, and 2-chloroadenosine were obtained from Sigma (St. Louis, MO). Western blots were performed using antibodies against α-tubulin (Cell Signaling, Danvers, MA), detyrosinated tubulin (Glu-tubulin; Millipore, Temecula, CA), sarcomeric actin, β-actin, β-myosin heavy chain (MHC), and β-tubulin (Sigma), and desmin and anti-cardiac MHC (Abcam, Cambridge, MA).

Statistics. Values are means ± SE. Statistical significance was defined as $P < 0.05$. Two-way ANOVA was used to test each variable for differences among the treatment groups with StatView (SAS Institute). If ANOVA demonstrated a significant effect, post hoc pairwise comparisons were made with Fisher's least significant difference test.

RESULTS

Adenosine or 2-chloroadenosine inhibits microtubule accumulation in cardiomyocytes. To examine the role of extracellular adenosine in cytoskeletal remodeling during hypertrophy, we analyzed cardiomyocyte cytoskeletal changes in response to a hypertrophic stimulus (PE) in the presence or absence of 2-chloroadenosine (a stable adenosine deaminase-resistant analog of adenosine). PE treatment for 48 h dramatically induced cytoskeletal remodeling, increasing the accumulation of sarcomeric proteins (sarcomeric actin), intermediate filaments (desmin), and microtubules (tubulin) in the Triton-insoluble cytoskeletal fraction while reducing microfilaments (β-actin; Fig. 1). Remarkably, 2-chloroadenosine treatment blocked the accumulation of tubulin in the cytoskeletal fraction by ~64% ($P < 0.01$) during the same period but only minimally affected other cytoskeletal elements. Similar results were demonstrated using adenosine (Fig. 1*B*). There was a slight (~17%) but significant ($P = 0.017$) reduction of PE-induced cytoskeletal sarcomeric actin in 2-chloroadenosine-treated cells, consistent with a role for microtubules as a scaffold for nascent myofilament formation (41).

2-Chloroadenosine or adenosine reduces stabilized microtubules. Net microtubule levels are determined by polymerization rate and stability. Detyrosination of α-tubulin is a posttranslational modification in which the COOH-terminal tyrosine residue is removed from α-tubulin [leaving a glutamine residue on the COOH terminus (Glu-tubulin)] (22). The carboxypeptidase, which mediates the loss of tyrosine, is predominantly active on polymerized tubulin (i.e., microtubules) (20), and detyrosination and other posttranslational modifications, such as α-tubulin acetylation, are increased on longer-lived microtubules (2). These modifications are commonly used to distinguish stable microtubules from more recently constructed or more dynamic microtubules (8). Immunofluorescent staining for α-tubulin revealed increased tubulin densification in PE-treated cells (Fig. 2*A*), which was reduced by 2-chloroadenosine treatment. Stabilized microtubules, identified with antibodies for Glu-tubulin and acetyl-tubulin, increased substan-

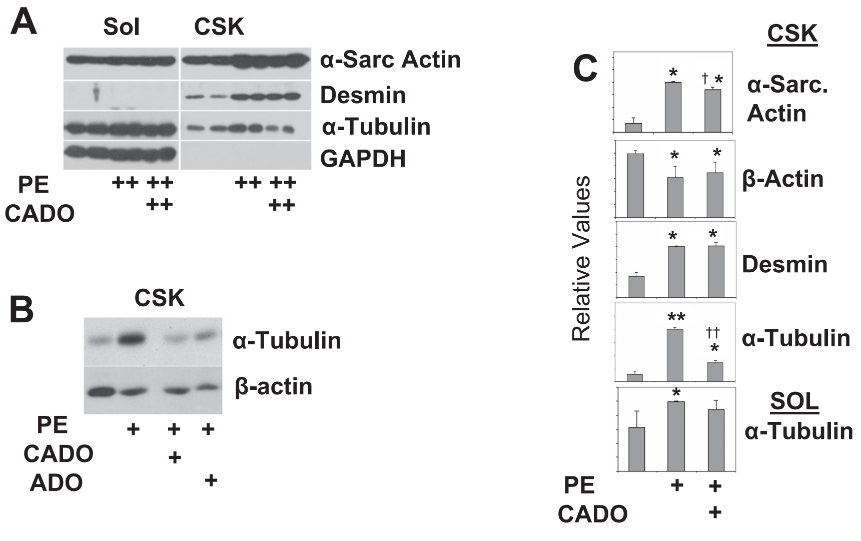

Fig. 1. 2-Chloroadenosine (CADO) or adenosine (ADO) reduces cytoskeletal tubulin. *A*: neonatal rat ventricular myocytes were treated with 50 μM phenylephrine (PE) in the presence or absence of 5 μM 2-chloroadenosine for 48 h. Cells were split into Triton-soluble (Sol) and insoluble [cytoskeletal (CSK)] fractions, separated on 9% polyacrylamide gel, and probed for α-sarcomeric actin, desmin, α-tubulin, and GAPDH. *B*: adenosine reduces cytoskeletal tubulin. Cells were treated with 50 μM PE + 10 μM adenosine + 1 μM erythro-9-(2-hydroxy-3-nonyl)adenine (EHNA) for 48 h. *C*: scanning densitometry was used to quantitate the level of expression of each protein in the different fractions (n = 8 for sarcomeric actin, n = 8 for β-actin, n = 2 for desmin, n = 12 for tubulin). †$P <$ 0.05 vs. PE. *$P \leq 0.05$ vs. control; **$P < 0.01$ vs. control; ††$P < 0.01$ vs. PE.

Downloaded from http://ajpheart.physiology.org/ at Univ of Minnesota on July 5, 2013

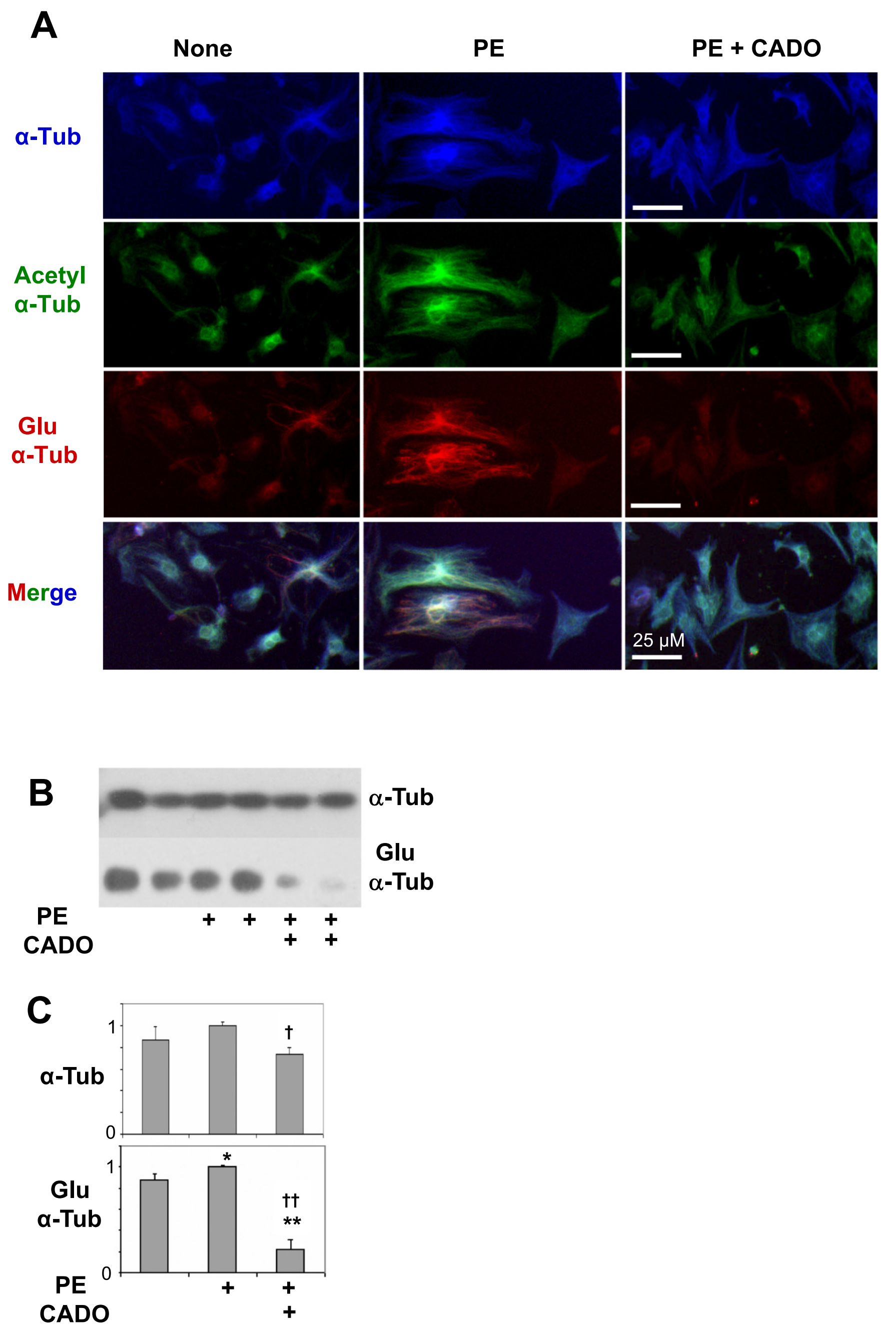

Fig. 2. 2-Chloroadenosine reduces levels of stabilized microtubules. *A*: cells were cultured for 48 h with 50 μM PE, fixed with methanol, and stained using antibodies for detyrosinated tubulin (Glu α-Tub, red), acetylated tubulin (Acetyl α-Tub, green), and total α-tubulin (α-Tub, blue), and stabilized microtubules were detected using fluorescent secondary antibodies. *B*: Western blot analysis of cytoskeletal α-tubulin and Glu-tubulin in confluent neonatal cardiomyocytes treated with 50 μM PE or PE + 5 μM 2-chloroadenosine. *C*: quantitation of Western blots (n = 6 per condition). $*P \leq 0.05$ vs. control. $\dagger P < 0.05$ vs. PE; $**P < 0.01$ vs. control; $\dagger\dagger P < 0.01$ vs. PE.

tially in response to PE and were much reduced after 2-chloroadenosine treatment, in agreement with the concept that 2-chloroadenosine reduces microtubule stability. Interestingly, microtubule detyrosination appeared particularly abundant in larger eccentric cells, whereas smaller cells often contained few, if any, stabilized microtubules (see supplemental Fig. 1, *A–C*, which shows larger field from Fig. 2*A*). Western blot analysis of cardiomyocytes confirmed that 2-chloroadenosine caused significant reductions of detyrosinated tubulin (see supplemental Fig. 2*A*). Furthermore, the reduction of cytoskeletal detyrosinated α-tubulin (~83%, $P < 0.01$) was greater than the reduction of cytoskeletal α-tubulin (~62%, $P < 0.01$) in the same cell lysates (see supplemental Fig. 2*B*), suggesting that 2-chloroadenosine has a specific effect on microtubule stability.

To distinguish between the effects of 2-chloroadenosine on cell growth and microtubule stability, cardiomyocytes were plated at higher density (1×10^5 cells/cm^2) on gelatin-coated dishes (conditions that promote cell spreading and confluency during the initial plating period). Under these conditions, microtubules were stabilized (showed increased detyrosination), persisted during serum withdrawal, and were only slightly increased in response to PE. In these cells, 48 h of PE + 2-chloroadenosine treatment reduced total microtubule levels by only 33% ($P < 0.01$) but reduced the level of detyrosinated tubulin by 80% ($P < 0.01$; Fig. 2, *B* and *C*). This suggests specific targeting of microtubule stabilization, rather than inhibition of microtubule polymerization, by 2-chloroadenosine. This is important, because stabilized microtubules are increased in response to pressure overload (44) and help drive changes in cellular morphology (19).

Downloaded from http://ajpheart.physiology.org/ at Univ of Minnesota on July 5, 2013

2-Chloroadenosine or adenosine selectively disrupts stabilized microtubules. We next examined whether 2-chloroadenosine or adenosine blocks detyrosination of newly formed microtubules, or whether it specifically disrupts established stabilized microtubules. Prehypertrophied cardiomyocytes (48 h of PE treatment) were briefly treated with 2-chloroadenosine or adenosine (under continued PE treatment), and stabilized microtubules (identified by Glu-tubulin antibody) were compared with the total microtubule population (identified by positive staining for tubulin). Colchicine was used as a control for nonselective microtubule depolymerization. Within 4 h, Glu-tubulin-containing microtubules were reduced in 2-chloroadenosine- or adenosine-treated cells but were still visible. As shown in Fig. 3, although stabilized microtubules (red) in control cells (PE alone) extended to the cell periphery, often to the same extent as total α-tubulin (green), Glu-tubulin-containing microtubules in 2-chloroadenosine- or adenosine-treated cells appeared retracted toward the center of the cell. At the same time, an extensive network of Glu-tubulin-negative microtubules (green) was still clearly evident after 2-chloroadenosine treatment. Colchicine also caused formation of broken and curled Glu-tubulin-containing microtubules. In contrast to 2-chloroadenosine or adenosine, colchicine disrupted the majority of microtubules but did not target stabilized microtubules. After 24 h, 2-chloroadenosine or adenosine had dramatically reduced Glu-tubulin levels but only moderately reduced total cytoskeletal α-tubulin levels (see supplemental Fig. 3*A*). By 48 h, 2-chloroadenosine reduced total cytoskeletal α-tubulin by 46% compared with PE-treated cells, and this was completely blocked by cotreatment with paclitaxel (see supplemental Fig. 3, *B* and *C*). This suggests that the stabilization and excess accumulation of microtubules in response to hypertrophy can be reversed by 2-chloroadenosine. The reduction of stabilized microtubules in prehypertrophied cells suggests that 2-chloroadenosine or adenosine does not inhibit microtubule stability simply by reducing cell growth. Blockade of the 2-chloroadenosine effect by paclitaxel implies that 2-chloroadenosine targets an endogenous microtubule stabilization mechanism but cannot reduce artificially stabilized microtubules.

2-Chloroadenosine, adenosine, and colchicine block hypertrophy. Because 2-chloroadenosine and adenosine have been shown to reduce cardiomyocyte hypertrophy and also reduce stabilization and accumulation of microtubules, we tested the hypothesis that reduction of microtubules would block hypertrophy. The PE-induced increase in cell area was prevented to a similar extent in cardiomyocytes treated with colchicine to depolymerize microtubules from 24–48 h of PE treatment and in cardiomyocytes treated with 2-chloroadenosine or adenosine (Fig. 4), demonstrating that microtubule polymerization plays a critical role in cell enlargement.

Together, the results in Figs. 1–4 demonstrate that hypertrophic stimuli increase microtubule accumulation and stabilization, that adenosine or 2-chloroadenosine treatment inhibits microtubule accumulation and stabilization, and that microtubule polymerization is necessary for cardiomyocyte hypertrophy.

Tubulin levels and stability are increased in CD73 KO mice under basal and pressure-overload conditions. Because treatment with 2-chloroadenosine or adenosine appeared to potently regulate microtubule stability and accumulation in cultured cardiomyocytes, we hypothesized that reduction of endogenous adenosine levels would increase microtubule stability and accumulation in vivo. In the adult mouse heart, increased microtubule accumulation during hypertrophy would be expected to exacerbate contractile dysfunction. Therefore, we investigated microtubule dynamics in CD73 KO mice, which are deficient in extracellular adenosine production. After 4 wk of TAC, contractile dysfunction and heart failure (indicated by increased lung weight-to-body weight ratio, LV dilation, and reduced ejection fraction) were significantly worse in CD73 KO than in WT mice (33) (Fig. 5*A*).

Using previously described methods to distinguish free from polymerized microtubules (55), we separated heart lysates into a free (containing unpolymerized tubulin) and a cold-released (generally thought to represent most microtubules) microtubule fraction and then further separated the cold-resistant insoluble pellet into a Triton-soluble (membrane) and a Triton-insoluble (remaining insoluble cytoskeleton, containing myofilaments, cold-stable microtubules, chromatin, etc.) fraction. Under basal conditions, levels of tubulin in the free and polymerized fractions tended to be higher in CD73 mice, although the difference was not significant. CD73 KO mice had higher levels of microtubule stability (identified by Glu-tubulin antibody) in the cold-released fraction, suggesting that microtubules were longer lived in CD73 KO mice. Pressure overload significantly increased the free and cold-released microtubule

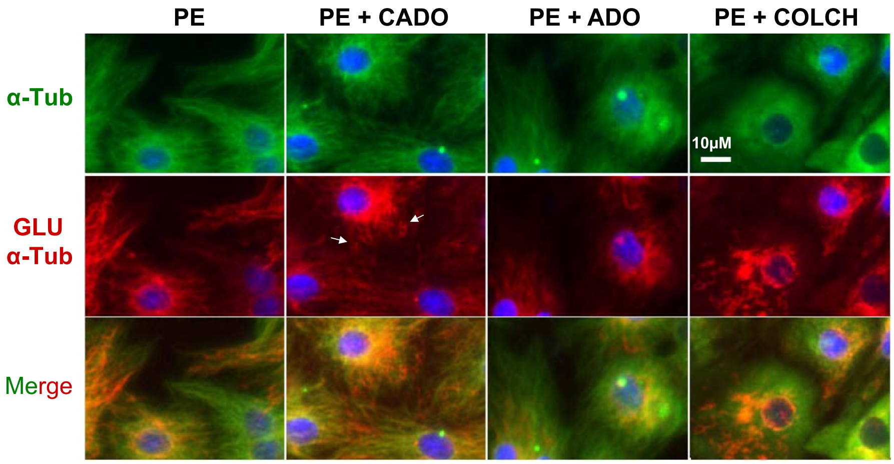

Fig. 3. 2-Chloroadenosine disrupts detyrosinated microtubules in hypertrophied neonatal rat ventricular myocytes. Cells were treated with 50 μM PE for 48 h and then for 4 h with PE in the presence or absence of 5 μM 2-chloroadenosine, 10 μM adenosine + 1 μM EHNA, or 100 nM colchicine (COLCH). Cells were stained using rabbit anti-Glu-tubulin followed by Alexa Fluor 555-labeled secondary (red) and FITC-labeled monoclonal (green) antibody to α-tubulin and Hoescht stain for DNA (blue).

Downloaded from http://ajpheart.physiology.org/ at Univ of Minnesota on July 5, 2013

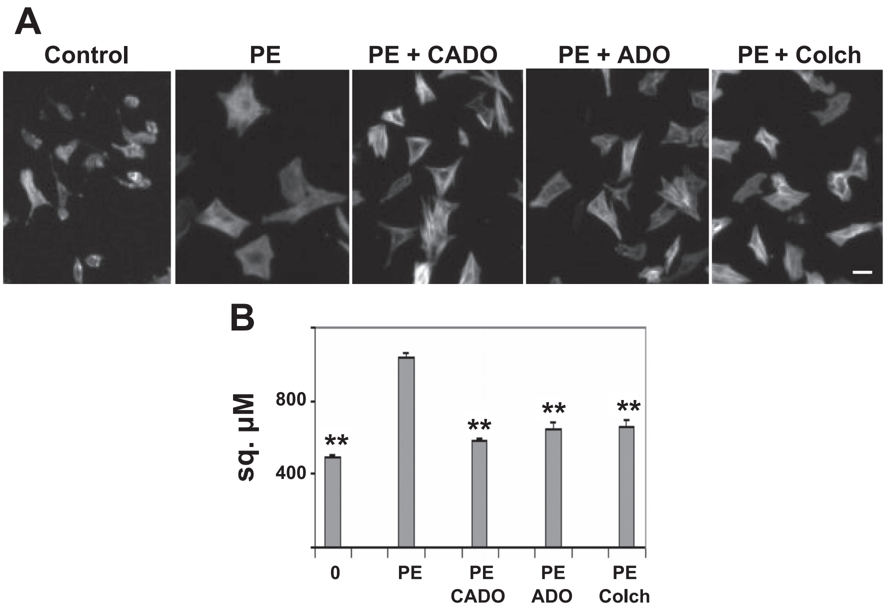

Fig. 4. Microtubules are required for cardiomyocyte hypertrophy. Cells were treated with 50 μM PE in the presence or absence of 5 μM 2-chloroadenosine or 10 μM adenosine + 1 μM EHNA for 48 h. PE-treated cells were also treated with 1 μM colchicine for 24–48 h to block polymerization of microtubules. *A*: cells were stained with rhodamine-phalloidin to measure cell area. *B*: at least 100 single cell areas were measured per condition. $^{**}P \leq 0.01$ vs. PE.

levels and stability in WT mice (Fig. 5, *B* and *C*), confirming previous studies using other animal models (27, 49, 55, 57). Free and cold-released microtubule levels and stability were also increased significantly in CD73 mice, and free tubulin was significantly higher in CD73 than in WT mice, whereas there was a trend toward increased microtubules ($P = 0.065$). Microtubule stability also trended higher in CD73 KO mice ($P = 0.061$).

Interestingly, the mice with the highest lung weights (indicating the most severe LV dysfunction) had less tubulin in the cold-released microtubule fraction than the mice from the same group with lower lung weights. Although most of the dynamic population of microtubules can be depolymerized by cold treatment, more stable microtubules are resistant to depolymerization by cold or chemical treatment (8, 9, 28, 44). Therefore, we examined the remaining pellets after cold treatment. Hearts from WT and CD73 KO mice had very little tubulin in the Triton-soluble membrane or insoluble cytoskeletal fractions of the remaining cold-resistant pellet under basal conditions. In response to pressure overload, WT and CD73 KO mice demonstrated significantly increased tubulin in the membrane fraction and the Triton-insoluble cytoskeletal fraction. Levels of tubulin and Glu-tubulin in these fractions were significantly higher in CD73 KO than in WT mice, consistent with the concept that higher adenosine levels in the WT mice reduced microtubule stabilization and accumulation in response to chronic pressure overload (Fig. 5, *B* and *C*). Interestingly, the levels of Triton-insoluble tubulin correlated strongly with the severity of congestive heart failure [as indicated by the lung weight-to-body weight ratio ($R = 0.802$) and inversely correlated with ejection fraction ($R = 0.809$); Fig. 5*D*], whereas no correlation was found for the levels of tubulin released by cold treatment. This suggests that microtubule stabilization, and perhaps interaction with other cytoskeletal proteins, may contribute more to the development of heart failure than simply the level of microtubules. There were no differences in the levels of sarcomeric actin, MHC, desmin, or β-actin in the cytoskeletal fraction between CD73 and WT mice. Expression of β-MHC, which is often activated by hypertrophic stimuli, was increased substantially in CD73 KO mice compared with WT mice, consistent with the greater hypertrophy (see supplemental Fig. 4).

Microtubules are required for hypertrophy and reduce contractile function during chronic pressure overload. Our results are in agreement with previous reports showing that pressure overload induces microtubule accumulation and stability (44, 48, 55). We also identified a correlation between cold-stable, Triton-insoluble, cytoskeletal tubulin levels and the degree of pulmonary congestion. Whether tubulin accumulated in the cytoskeleton as a result of hypertrophy and heart failure or whether it contributed to heart failure was unclear. To determine the contribution of cardiac microtubules to hypertrophy and heart failure, we used Balb/c mice, which more rapidly develop dilation and heart failure following TAC but develop less hypertrophy than the 129 strain of mice (the background of the CD73 KO mice). Balb/c mice were treated with colchicine every other day beginning 3 days after TAC or sham surgery, and treatment was stopped 2 days before ejection fraction was determined and tissue was collected (a total of 2 wk of TAC). In agreement with a pivotal role of microtubule accumulation in progression of heart failure in response to systolic overload, hypertrophy was significantly reduced and LV function was significantly enhanced in mice treated with colchicine compared with saline-treated mice (Table 1).

Analysis of Triton X-100-insoluble lysates (cytoskeletal fractions) from these hearts demonstrated again that TAC increased α-tubulin accumulation and detyrosination in cardiac tissue, whereas colchicine reduced the levels of insoluble tubulin and detyrosination as expected (Fig. 6). Colchicine also reduced total tubulin levels, consistent with a downregulation of tubulin synthesis by high levels of free tubulin, as previously described (18, 37). At the same time, colchicine had little effect on sarcomeric actin, desmin, or cadherin levels, but it did reduce β-actin levels in the mice exposed to TAC (see supplemental Fig. 5). This reduction of β-actin was not found in nonbanded mice treated with colchicine. Interestingly, colchicine alone did increase β-MHC levels ($P < 0.001$) and further increased the expression of β-MHC after TAC ($P = 0.048$; see

Downloaded from http://ajpheart.physiology.org/ at Univ of Minnesota on July 5, 2013

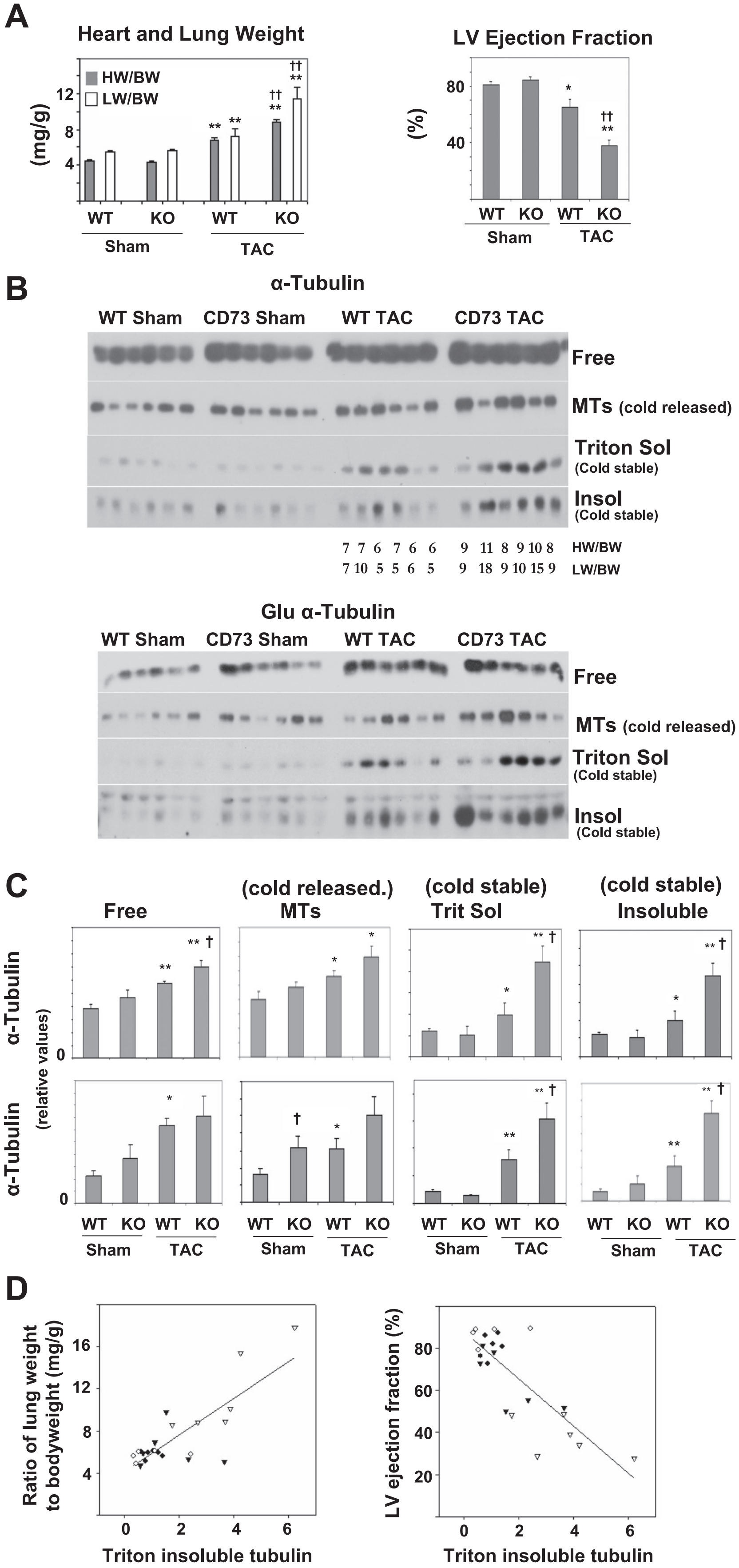

Fig. 5. Cardiac function is impaired and microtubule (MT) stability is increased in adenosine-deficient mice. *A*: CD73 deletion (KO) exacerbates hypertrophy and dilation and worsens contractile function in response to transverse aortic constriction (TAC). *B*: fractions from ventricular homogenates of wild-type (WT) and CD73 KO mice 4 wk after sham or TAC surgery were analyzed by Western blot using antibody against total α-tubulin or Glu-tubulin. HW/BW, heart weight-to-body weight ratio; LW/BW, lung weight-to-body weight ratio. *C*: quantitation of Western blots. $*P \leq 0.05$; $**P \leq 0.01$ vs. sham control. $\dagger P < 0.05$; $\dagger\dagger P < 0.01$ vs. WT animals with the same surgery. *D*: cold-stable Triton-insoluble tubulin levels correlate positively with congestive heart failure (as indicated by lung weight-to-body weight ratio) and inversely with ejection fraction. ▽, CD73 KO TAC; ▼, WT TAC; ○, CD73 KO sham; ●, WT sham. LV, left ventricle.

Downloaded from http://ajpheart.physiology.org/ at Univ of Minnesota on July 5, 2013

Table 1. *Colchicine improves contractile function during pressure overload*

	LVESD, mm	LVEDD, mm	EF, %	HR, beats/min	LW/BW	HW/BW
Saline	2.7±0.4	4.0±0.4	68.3±-5.6	500±36	5.7±0.1	4.1±0.2
Colchicine	2.2±0.3†	3.4±0.4†	73.9±4.0†	474±18	5.8±0.6	4.1±0.1
TAC + saline	3.6±0.1*	4.4±0.1*	43.6±3.7*	489±51	9.5±2.9*	5.4±0.7*
TAC + colchicine	3.0±0.1†*	3.9±0.04†*	55±3.0†*	438±59	6.7±0.9†	4.5±0.68†

Values are means ± SE. LVESD and LVEDD, left ventricular end-systolic and end-diastolic diameter; EF, ejection fraction; HR, heart rate; LW/BW, lung weight-to-body weight ratio; HW/BW, heart weight-to-body weight ratio; TAC, transverse aortic constriction. *Significantly different ($P < 0.05$) from sham (i.e., no TAC) with same drug or vehicle treatment. †Significantly different ($P < 0.05$) from saline-treated mice with same surgical procedure.

supplemental Fig. 5). The activation of mammalian target of rapamycin/70-kDa S6 kinase and ERK by TAC also was not inhibited by colchicine (data not shown), suggesting that the hypertrophic signaling response to pressure overload was still intact, but the downstream structural changes associated with microtubule accumulation and dysfunction were blocked specifically by inhibition of microtubule polymerization. These results confirm that microtubule stabilization plays a role in progression of hypertrophy and cardiac dysfunction during pressure overload and support the role of adenosine in protecting the heart by reducing cardiac microtubule stabilization.

DISCUSSION

It has long been recognized that adenosine exerts cardioprotective effects in the preconditioning response. Here we identify a novel role for adenosine in the heart as a potent regulator of the cardiomyocyte microtubule cytoskeleton during hypertrophy. Microtubules increase during pressure-overload hypertrophy and during hypertrophy of cultured cells. Adenosine treatment reduced the accumulation of microtubules in vitro, whereas reduction of adenosine production by genetic deletion of CD73 increased cardiac microtubule accumulation and stability in vivo. Data from studies using microtubule-inhibiting drugs suggest that microtubule polymerization is required for hypertrophic growth (52, 54, 59), so it is possible that the effects of adenosine on microtubule stability may limit hypertrophy. However, the maladaptive densification of microtubules in response to pressure overload does not necessarily promote hypertrophy (48, 55). In fact, in response to LV pressure overload, dogs that exhibited increased microtubule densification developed less hypertrophy than dogs that maintained normal microtubule density (48). However, although the animals with normal microtubule density exhibited compensatory hypertrophy with preserved ejection fraction, the dogs with increased microtubule density developed ventricular dilation and heart failure. In support of a role for microtubules in driving maladaptive ventricular remodeling, we show that colchicine treatment reduced ventricular dilation and improved contractile function during chronic pressure overload. Recently, Cheng et al. (10) used mutant β-tubulin isoforms that differently altered microtubule stability to more elegantly test the hypothesis that microtubule stabilization contributes to cardiac dysfunction during hypertrophy. Destabilizing mutations improved contractile function during pressure overload-induced hypertrophy but did not reduce hypertrophy, whereas stabilizing mutations impaired ventricular function and increased cardiac hypertrophy, even under basal conditions (10).

Adenosine regulation of microtubule stability. The finding that detyrosinated α-tubulin levels and cold-stable α-tubulin are increased in CD73 KO mice exposed to pressure overload suggests that decreased adenosine availability leads to increased microtubule stability. Interestingly, even brief treatment with 2-chloroadenosine or adenosine appeared to promote disassembly of stabilized microtubules compared with control cultures (Fig. 4), whereas prolonged treatment reduced levels of Glu-tubulin-containing microtubules to a much greater degree than total microtubules. How 2-chloroadenosine or adenosine might target stabilized microtubules is not clear. There is evidence that inhibition of protein phosphatase 2A also causes selective breakdown of stabilized microtubules in fibroblasts and epithelial cells (21). Interestingly, myocardial protein phosphatase 2A activity is increased by activation of the adenosine A_1 receptor but decreased by activation of the adenosine A_{2A} receptor (53). Our studies have not identified a specific adenosine receptor subtype agonist that reproduces the effects of 2-chloroadenosine or adenosine (data not shown). The role of adenosine receptors in this regard is likely to be complex, inasmuch as overexpression of adenosine A_1 (14) or

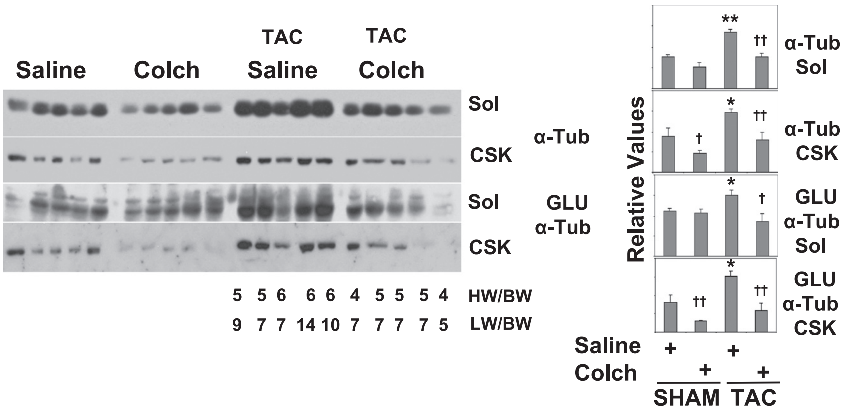

Fig. 6. Colchicine reduces pressure overload-induced accumulation of cytoskeletal tubulin. *Left*: after 2 wk of TAC, mice treated with saline or colchicine were killed, and heart tissue was fractionated into Triton-soluble and -insoluble (cytoskeletal) fractions and analyzed by Western blot. α-Tubulin (*top*) and Glu-α-tubulin (*bottom*) increased significantly in response to TAC, and these increases were blocked by colchicine treatment. *C*: quantitation of Western blot. *$P \leq 0.05$; **$P \leq 0.01$ vs. sham control. †$P < 0.05$; ††$P < 0.01$ vs. saline-treated animals with the same surgery.

Downloaded from http://ajpheart.physiology.org/ at Univ of Minnesota on July 5, 2013

A_3 (4) receptor increases dilation and heart failure, whereas knockout of adenosine A_1 receptor does not exacerbate hypertrophy and heart failure, and knockout of adenosine A_3 receptor actually protects the heart against hypertrophy and remodeling during pressure overload (33). This suggests a nonreceptor-mediated mechanism, a cooperative effect of multiple adenosine receptors, or a previously unrecognized adenosine-binding receptor that mediates the effects of adenosine on microtubule dynamics.

Effects of microtubules. Although there is evidence that microtubules can mechanically impair contractile function in isolated hypertrophied cardiomyocytes (50, 55) and in vivo (29), there was no correlation between cold-released microtubules and the degree of pulmonary congestion in hearts from the CD73 KO mice in the present study. Interestingly, however, the level of cold-resistant Triton-insoluble tubulin associated with the cytoskeletal fraction correlated significantly with pulmonary congestion and inversely correlated with ejection fraction. This fraction represents <5% of the total tubulin, whereas the cold-released microtubules represent ~30–40% of total tubulin. Sarcomeric actin and myosin make up the majority of the Triton-insoluble fraction, and desmin and β-actin are also most abundant in this fraction. It is possible that the increase of tubulin in this fraction, as well as in the cold-treated membrane fraction, represents increased linkages between microtubules and Triton-insoluble cytoskeletal or membrane elements, which may directly interfere with contractile performance by increasing cell stiffness. There is also evidence that excessive microtubule stabilization has adverse effects in cardiomyocytes in ways other than mechanical impairment of contractile performance. For example, microtubule stabilization can increase NADPH-dependent oxidative stress (1, 12) and promote nuclear translocation of p53, increasing sensitivity to apoptosis (17). Stable microtubules have also been found to play a role in streptozotocin-induced cardiac dysfunction in the diabetic rat (46). Furthermore, microtubule-associated protein 4 decoration of microtubules [which can stabilize microtubules (51) and increases in response to pressure overload (44)] has been shown to reduce mRNA trafficking along microtubules in cardiomyocytes (45), suggesting that this modification may reduce myofibril-localized translation of sarcomeric proteins and impair compensatory growth, which helps normalize wall stress during pressure overload. It will be interesting in this regard to determine whether adenosine regulates microtubule stability through modulation of microtubule-associated protein 4 activity.

In summary, we have identified a novel role for extracellular adenosine in regulating cardiomyocyte microtubule dynamics. Because interstitial adenosine levels have been shown to fall as compensatory hypertrophy progresses to heart failure (15, 35), it is possible that adenosine plays a role in delaying this transition by reducing stabilization and accumulation of microtubules.

Limitations. Although neonatal cardiomyocytes are frequently used to investigate hypertrophic signaling pathways, we recognize that they are structurally and physiologically different from adult cardiomyocytes. In addition to differences in calcium handling, neonatal cardiomyocytes have a less dense sarcomeric cytoskeleton than adult cardiomyocytes, and cell shape and contractile properties of neonatal cardiomyocytes are different from those of adult cardiomyocytes. These differences are likely to affect microtubule dynamics, as well as the influence of microtubules on cell size and shape. Another limitation of this study is that 2-chloroadenosine, rather than the natural occurring nucleoside adenosine, was predominantly used on cells. 2-Chloroadenosine was chosen, because adenosine is rapidly degraded to inosine by adenosine deaminase, and although EHNA (the most common adenosine deaminase inhibitor) can be used to prevent adenosine degradation, EHNA has undesired nonspecific effects, including inhibition of dynein (microtubule motor protein) (6) and multiple phosphodiesterases (23, 34).

GRANTS

This study was supported by National Heart, Lung, and Blood Institute Grants HL-21872 and HL-71790 and American Heart Association Research Grants 0330136N and 0160275Z. J. Fassett is the recipient of a Scientist Development Award from the American Heart Association.

REFERENCES

1. **Alexandre J, Hu Y, Lu W, Pelicano H, Huang P.** Novel action of paclitaxel against cancer cells: bystander effect mediated by reactive oxygen species. *Cancer Res* 67: 3512–3517, 2007.
2. **Barra HS, Arce CA, Argarana CE.** Posttranslational tyrosination/detyrosination of tubulin. *Mol Neurobiol* 2: 133–153, 1988.
3. **Bifulco M, Laezza C, Stingo S, Wolff J.** 2′,3′-Cyclic nucleotide 3′-phosphodiesterase: a membrane-bound, microtubule-associated protein and membrane anchor for tubulin. *Proc Natl Acad Sci USA* 99: 1807–1812, 2002.
4. **Black RG Jr, Guo Y, Ge ZD, Murphree SS, Prabhu SD, Jones WK, Bolli R, Auchampach JA.** Gene dosage-dependent effects of cardiac-specific overexpression of the A_3 adenosine receptor. *Circ Res* 91: 165–172, 2002.
5. **Bloom K.** Microtubule cytoskeleton: navigating the intracellular landscape. *Curr Biol* 13: R430–R432, 2003.
6. **Bouchard P, Penningroth SM, Cheung A, Gagnon C, Bardin CW.** Erythro-9-[3-(2-hydroxynonyl)]adenine is an inhibitor of sperm motility that blocks dynein ATPase and protein carboxylmethylase activities. *Proc Natl Acad Sci USA* 78: 1033–1036, 1981.
7. **Brangwynne CP, MacKintosh FC, Kumar S, Geisse NA, Talbot J, Mahadevan L, Parker KK, Ingber DE, Weitz DA.** Microtubules can bear enhanced compressive loads in living cells because of lateral reinforcement. *J Cell Biol* 173: 733–741, 2006.
8. **Bre MH, Kreis TE, Karsenti E.** Control of microtubule nucleation and stability in Madin-Darby canine kidney cells: the occurrence of noncentrosomal, stable detyrosinated microtubules. *J Cell Biol* 105: 1283–1296, 1987.
9. **Cambray-Deakin MA, Burgoyne RD.** Acetylated and detyrosinated α-tubulins are co-localized in stable microtubules in rat meningeal fibroblasts. *Cell Motil Cytoskeleton* 8: 284–291, 1987.
10. **Cheng G, Zile MR, Takahashi M, Baicu CF, Bonnema DD, Cabral F, Menick DR, Cooper G.** A direct test of the hypothesis that increased microtubule network density contributes to contractile dysfunction of the hypertrophied heart. *Am J Physiol Heart Circ Physiol* 294: H2231–H2241, 2008.
11. **Cooper GT.** Cardiocyte cytoskeleton in hypertrophied myocardium. *Heart Fail Rev* 5: 187–201, 2000.
12. **Devillard L, Vandroux D, Tissier C, Brochot A, Voisin S, Rochette L, Athias P.** Tubulin ligands suggest a microtubule-NADPH oxidase relationship in postischemic cardiomyocytes. *Eur J Pharmacol* 548: 64–73, 2006.
13. **Donato M, Gelpi RJ.** Adenosine and cardioprotection during reperfusion—an overview. *Mol Cell Biochem* 251: 153–159, 2003.
14. **Funakoshi H, Chan TO, Good JC, Libonati JR, Piuhola J, Chen X, MacDonnell SM, Lee LL, Herrmann DE, Zhang J, Martini J, Palmer TM, Sanbe A, Robbins J, Houser SR, Koch WJ, Feldman AM.** Regulated overexpression of the A_1-adenosine receptor in mice results in adverse but reversible changes in cardiac morphology and function. *Circulation* 114: 2240–2250, 2006.
15. **Funakoshi H, Zacharia LC, Tang Z, Zhang J, Lee LL, Good JC, Herrmann DE, Higuchi Y, Koch WJ, Jackson EK, Chan TO, Feldman**

Downloaded from http://ajpheart.physiology.org/ at Univ of Minnesota on July 5, 2013

AM. A_1 adenosine receptor upregulation accompanies decreasing myocardial adenosine levels in mice with left ventricular dysfunction. *Circulation* 115: 2307–2315, 2007.
16. **Gan XT, Rajapurohitam V, Haist JV, Chidiac P, Cook MA, Karmazyn M.** Inhibition of phenylephrine-induced cardiomyocyte hypertrophy by activation of multiple adenosine receptor subtypes. *J Pharmacol Exp Ther* 312: 27–34, 2005.
17. **Giannakakou P, Nakano M, Nicolaou KC, O'Brate A, Yu J, Blagosklonny MV, Greber UF, Fojo T.** Enhanced microtubule-dependent trafficking and p53 nuclear accumulation by suppression of microtubule dynamics. *Proc Natl Acad Sci USA* 99: 10855–10860, 2002.
18. **Gong ZY, Brandhorst B.** Autogenous regulation of tubulin synthesis via RNA stability during sea urchin embryogenesis. *Development* 102: 31–43, 1988.
19. **Gundersen GG, Khawaja S, Bulinski JC.** Generation of a stable, posttranslationally modified microtubule array is an early event in myogenic differentiation. *J Cell Biol* 109: 2275–2288, 1989.
20. **Gundersen GG, Khawaja S, Bulinski JC.** Postpolymerization detyrosination of α-tubulin: a mechanism for subcellular differentiation of microtubules. *J Cell Biol* 105: 251–264, 1987.
21. **Gurland G, Gundersen GG.** Protein phosphatase inhibitors induce the selective breakdown of stable microtubules in fibroblasts and epithelial cells. *Proc Natl Acad Sci USA* 90: 8827–8831, 1993.
22. **Hallak ME, Rodriguez JA, Barra HS, Caputto R.** Release of tyrosine from tyrosinated tubulin. Some common factors that affect this process and the assembly of tubulin. *FEBS Lett* 73: 147–150, 1977.
23. **Haynes J Jr, Killilea DW, Peterson PD, Thompson WJ.** Erythro-9-(2-hydroxy-3-nonyl)adenine inhibits cyclic-3′,5′-guanosine monophosphate-stimulated phosphodiesterase to reverse hypoxic pulmonary vasoconstriction in the perfused rat lung. *J Pharmacol Exp Ther* 276: 752–757, 1996.
24. **Hu P, Zhang D, Swenson L, Chakrabarti G, Abel ED, Litwin SE.** Minimally invasive aortic banding in mice: effects of altered cardiomyocyte insulin signaling during pressure overload. *Am J Physiol Heart Circ Physiol* 285: H1261–H1269, 2003.
25. **Ingber DE.** Mechanical signaling and the cellular response to extracellular matrix in angiogenesis and cardiovascular physiology. *Circ Res* 91: 877–887, 2002.
26. **Ingber DE.** Tensegrity. I. Cell structure and hierarchical systems biology. *J Cell Sci* 116: 1157–1173, 2003.
27. **Ishibashi Y, Tsutsui H, Yamamoto S, Takahashi M, Imanaka-Yoshida K, Yoshida T, Urabe Y, Sugimachi M, Takeshita A.** Role of microtubules in myocyte contractile dysfunction during cardiac hypertrophy in the rat. *Am J Physiol Heart Circ Physiol* 271: H1978–H1987, 1996.
28. **Khawaja S, Gundersen GG, Bulinski JC.** Enhanced stability of microtubules enriched in detyrosinated tubulin is not a direct function of detyrosination level. *J Cell Biol* 106: 141–149, 1988.
29. **Koide M, Hamawaki M, Narishige T, Sato H, Nemoto S, DeFreyte G, Zile MR, Cooper GI, Carabello BA.** Microtubule depolymerization normalizes in vivo myocardial contractile function in dogs with pressure-overload left ventricular hypertrophy. *Circulation* 102: 1045–1052, 2000.
30. **Lankford AR, Yang JN, Rose'Meyer R, French BA, Matherne GP, Fredholm BB, Yang Z.** Effect of modulating cardiac A_1 adenosine receptor expression on protection with ischemic preconditioning. *Am J Physiol Heart Circ Physiol* 290: H1469–H1473, 2006.
31. **Liao Y, Takashima S, Asano Y, Asakura M, Ogai A, Shintani Y, Minamino T, Asanuma H, Sanada S, Kim J, Ogita H, Tomoike H, Hori M, Kitakaze M.** Activation of adenosine A_1 receptor attenuates cardiac hypertrophy and prevents heart failure in murine left ventricular pressure-overload model. *Circ Res* 93: 759–766, 2003.
32. **Lopez de Heredia M, Jansen RP.** mRNA localization and the cytoskeleton. *Curr Opin Cell Biol* 16: 80–85, 2004.
33. **Lu Z, Fassett J, Xu X, Hu X, Zhu G, French J, Zhang P, Schnermann J, Bache RJ, Chen Y.** Adenosine A_3 receptor deficiency exerts unanticipated protective effects on the pressure-overloaded left ventricle. *Circulation* 118: 1713–1721, 2008.
34. **Mery PF, Pavoine C, Pecker F, Fischmeister R.** Erythro-9-(2-hydroxy-3-nonyl)adenine inhibits cyclic GMP-stimulated phosphodiesterase in isolated cardiac myocytes. *Mol Pharmacol* 48: 121–130, 1995.
35. **Meyer TE, Chung ES, Perlini S, Norton GR, Woodiwiss AJ, Lorbar M, Fenton RA, Dobson JG Jr.** Antiadrenergic effects of adenosine in pressure overload hypertrophy. *Hypertension* 37: 862–868, 2001.
36. **Mubagwa K, Flameng W.** Adenosine, adenosine receptors and myocardial protection: an updated overview. *Cardiovasc Res* 52: 25–39, 2001.
37. **Pachter JS, Yen TJ, Cleveland DW.** Autoregulation of tubulin expression is achieved through specific degradation of polysomal tubulin mRNAs. *Cell* 51: 283–292, 1987.
38. **Palazzo AF, Cook TA, Alberts AS, Gundersen GG.** mDia mediates Rho-regulated formation and orientation of stable microtubules. *Nat Cell Biol* 3: 723–729, 2001.
39. **Palmer KJ, Watson P, Stephens DJ.** The role of microtubules in transport between the endoplasmic reticulum and Golgi apparatus in mammalian cells. *Biochem Soc Symp* 1–13, 2005.
40. **Peart J, Headrick JP.** Adenosine-mediated early preconditioning in mouse: protective signaling and concentration dependent effects. *Cardiovasc Res* 58: 589–601, 2003.
41. **Pizon V, Gerbal F, Diaz CC, Karsenti E.** Microtubule-dependent transport and organization of sarcomeric myosin during skeletal muscle differentiation. *EMBO J* 24: 3781–3792, 2005.
42. **Reynolds IJ, Rintoul GL.** Mitochondrial stop and go: signals that regulate organelle movement. *Sci STKE* 2004: PE46, 2004.
43. **Rodriguez OC, Schaefer AW, Mandato CA, Forscher P, Bement WM, Waterman-Storer CM.** Conserved microtubule-actin interactions in cell movement and morphogenesis. *Nat Cell Biol* 5: 599–609, 2003.
44. **Sato H, Nagai T, Kuppuswamy D, Narishige T, Koide M, Menick DR, Cooper GT.** Microtubule stabilization in pressure overload cardiac hypertrophy. *J Cell Biol* 139: 963–973, 1997.
45. **Scholz D, Baicu CF, Tuxworth WJ, Xu L, Kasiganesan H, Menick DR, Cooper GT.** Microtubule-dependent distribution of mRNA in adult cardiocytes. *Am J Physiol Heart Circ Physiol* 294: H1135–H1144, 2008.
46. **Shiels H, O'Connell A, Qureshi MA, Howarth FC, White E, Calaghan S.** Stable microtubules contribute to cardiac dysfunction in the streptozotocin-induced model of type 1 diabetes in the rat. *Mol Cell Biochem* 294: 173–180, 2007.
47. **Siegrist SE, Doe CQ.** Microtubule-induced cortical cell polarity. *Genes Dev* 21: 483–496, 2007.
48. **Tagawa H, Koide M, Sato H, Zile MR, Carabello BA, Cooper GT.** Cytoskeletal role in the transition from compensated to decompensated hypertrophy during adult canine left ventricular pressure overloading. *Circ Res* 82: 751–761, 1998.
49. **Tagawa H, Rozich JD, Tsutsui H, Narishige T, Kuppuswamy D, Sato H, McDermott PJ, Koide M, Cooper GT.** Basis for increased microtubules in pressure-hypertrophied cardiocytes. *Circulation* 93: 1230–1243, 1996.
50. **Tagawa H, Wang N, Narishige T, Ingber DE, Zile MR, Cooper GT.** Cytoskeletal mechanics in pressure-overload cardiac hypertrophy. *Circ Res* 80: 281–289, 1997.
51. **Takahashi M, Shiraishi H, Ishibashi Y, Blade KL, McDermott PJ, Menick DR, Kuppuswamy D, Cooper GT.** Phenotypic consequences of β_1-tubulin expression and MAP4 decoration of microtubules in adult cardiocytes. *Am J Physiol Heart Circ Physiol* 285: H2072–H2083, 2003.
52. **Takahashi M, Tsutsui H, Tagawa H, Igarashi-Saito K, Imanaka-Yoshida K, Takeshita A.** Microtubules are involved in early hypertrophic responses of myocardium during pressure overload. *Am J Physiol Heart Circ Physiol* 275: H341–H348, 1998.
53. **Tikh EI, Fenton RA, Chen JF, Schwarzschild MA, Dobson JG Jr.** Adenosine A_1 and A_{2A} receptor regulation of protein phosphatase 2A in the murine heart. *J Cell Physiol* 216: 83–90, 2008.
54. **Tsutsui H, Ishibashi Y, Takahashi M, Namba T, Tagawa H, Imanaka-Yoshida K, Takeshita A.** Chronic colchicine administration attenuates cardiac hypertrophy in spontaneously hypertensive rats. *J Mol Cell Cardiol* 31: 1203–1213, 1999.
55. **Tsutsui H, Ishihara K, Cooper GT.** Cytoskeletal role in the contractile dysfunction of hypertrophied myocardium. *Science* 260: 682–687, 1993.
56. **Wang N, Naruse K, Stamenovic D, Fredberg JJ, Mijailovich SM, Tolic-Norrelykke IM, Polte T, Mannix R, Ingber DE.** Mechanical behavior in living cells consistent with the tensegrity model. *Proc Natl Acad Sci USA* 98: 7765–7770, 2001.
57. **Wang X, Li F, Campbell SE, Gerdes AM.** Chronic pressure overload cardiac hypertrophy and failure in guinea pigs. II. Cytoskeletal remodeling. *J Mol Cell Cardiol* 31: 319–331, 1999.
58. **Xu X, Fassett J, Hu X, Zhu G, Lu Z, Li Y, Schnermann J, Bache RJ, Chen Y.** Ecto-5′-nucleotidase deficiency exacerbates pressure-overload-induced left ventricular hypertrophy and dysfunction. *Hypertension* 51: 1557–1564, 2008.

Downloaded from http://ajpheart.physiology.org/ at Univ of Minnesota on July 5, 2013

59. **Yutao X, Geru W, Xiaojun B, Tao G, Aiqun M.** Mechanical stretch-induced hypertrophy of neonatal rat ventricular myocytes is mediated by β_1-integrin-microtubule signaling pathways. *Eur J Heart Fail* 8: 16–22, 2006.
60. **Zhang P, Xu X, Hu X, van Deel ED, Zhu G, Chen Y.** Inducible nitric oxide synthase deficiency protects the heart from systolic overload-induced ventricular hypertrophy and congestive heart failure. *Circ Res* 100: 1089–1098, 2007.
61. **Zhang W, Anger T, Su J, Hao J, Xu X, Zhu M, Gach A, Cui L, Liao R, Mende U.** Selective loss of fine tuning of $G_{q/11}$ signaling by RGS2 protein exacerbates cardiomyocyte hypertrophy. *J Biol Chem* 281: 5811–5820, 2006.

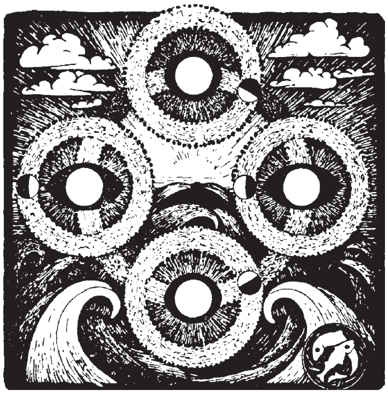

Downloaded from http://ajpheart.physiology.org/ at Univ of Minnesota on July 5, 2013
