# Supplementary material for: Microtubule Actin Cross-Linking Factor 1 Regulates Cardiomyocyte Microtubule Distribution and Adaptation to Hemodynamic Overload
Source: PLoS One. 2013 Sep 26;8(9):e73887. doi: 10.1371/journal.pone.0073887 (PMC3784444; doi:10.1371/journal.pone.0073887)
Supplement: File S2 — Previous manuscripts from which some parts of the data in figure 5 were obtained. (PDF) [file pone.0073887.s002.pdf]

AMPK attenuates microtubule proliferation in cardiac hypertrophy

John T. Fassett, Xinli Hu, Xin Xu, Zhongbing Lu, Ping Zhang, Yingjie Chen and Robert J. Bache

Am J Physiol Heart Circ Physiol 304:H749-H758, 2013. First published 11 January 2013;
doi: 10.1152/ajpheart.00935.2011

You might find this additional info useful...

This article cites 45 articles, 31 of which you can access for free at:
http://ajpheart.physiology.org/content/304/5/H749.full#ref-list-1

Updated information and services including high resolution figures, can be found at:
http://ajpheart.physiology.org/content/304/5/H749.full

Additional material and information about ***American Journal of Physiology - Heart and Circulatory Physiology*** can be found at:
http://www.the-aps.org/publications/ajpheart

This information is current as of July 5, 2013.

American Journal of Physiology - Heart and Circulatory Physiology publishes original investigations on the physiology of the heart, blood vessels, and lymphatics, including experimental and theoretical studies of cardiovascular function at all levels of organization ranging from the intact animal to the cellular, subcellular, and molecular levels. It is published 24 times a year (twice monthly) by the American Physiological Society, 9650 Rockville Pike, Bethesda MD 20814-3991. Copyright © 2013 the American Physiological Society. ESSN: 1522-1539. Visit our website at http://www.the-aps.org/.

Downloaded from http://ajpheart.physiology.org/ at Univ of Minnesota on July 5, 2013

Am J Physiol Heart Circ Physiol 304: H749–H758, 2013.
First published January 11, 2013; doi:10.1152/ajpheart.00935.2011.

AMPK attenuates microtubule proliferation in cardiac hypertrophy

John T. Fassett,[1] Xinli Hu,[2] Xin Xu,[1] Zhongbing Lu,[2] Ping Zhang,[1] Yingjie Chen,[1,2] and Robert J. Bache[1]
[1]*Cardiovascular Division and* [2]*Vascular Biology Center, Department of Medicine, University of Minnesota Medical School, Minneapolis, Minnesota*

Submitted 20 September 2011; accepted in final form 19 December 2012

Fassett JT, Hu X, Xu X, Lu Z, Zhang P, Chen Y, Bache RJ. AMPK attenuates microtubule proliferation in cardiac hypertrophy. *Am J Physiol Heart Circ Physiol* 304: H749–H758, 2013. First published January 11, 2013; doi:10.1152/ajpheart.00935.2011.—Cell hypertrophy requires increased protein synthesis and expansion of the cytoskeletal networks that support cell enlargement. AMPK limits anabolic processes, such as protein synthesis, when energy supply is insufficient, but its role in cytoskeletal remodeling is not known. Here, we examined the influence of AMPK in cytoskeletal remodeling during cardiomyocyte hypertrophy, a clinically relevant condition in which cardiomyocytes enlarge but do not divide. In neonatal cardiomyocytes, activation of AMPK with 5-aminoimidazole carboxamide ribonucleotide (AICAR) or expression of constitutively active AMPK (CA-AMPK) attenuated cell area increase by hypertrophic stimuli (phenylephrine). AMPK activation had little effect on intermediate filaments or myofilaments but dramatically reduced microtubule stability, as measured by detyrosinated tubulin levels and cytoskeletal tubulin accumulation. Importantly, low-level AMPK activation limited cell expansion and microtubule growth independent of mTORC1 or protein synthesis repression, identifying a new mechanism by which AMPK regulates cell growth. Mechanistically, AICAR treatment increased Ser-915 phosphorylation of microtubule-associated protein 4 (MAP4), which reduces affinity for tubulin and prevents stabilization of microtubules (MTs). RNAi knockdown of MAP4 confirmed its critical role in cardiomyocyte MT stabilization. In support of a pathophysiological role for AMPK regulation of cardiac microtubules, AMPK α_2 KO mice exposed to pressure overload (transverse aortic constriction; TAC) demonstrated reduced MAP4 phosphorylation and increased microtubule accumulation that correlated with the severity of contractile dysfunction. Together, our data identify the microtubule cytoskeleton as a sensitive target of AMPK activity, and the data suggest a novel role for AMPK in limiting accumulation and densification of microtubules that occurs in response to hypertrophic stress.

AMP-activated protein kinase; cytoskeleton; hypertrophy; microtubule-associated protein 4; microtubule

MICROTUBULES (MTS) ARE DYNAMIC filamentous elements, which regulate cell size, morphology, and subcellular organization. Microtubules act as structural elements that resist actomyosin contractility (36) and also serve as tracks for transport of mRNA (25), proteins (16, 32), and organelles (17, 42). Although microtubules play important roles in many cell functions, stabilization and aberrant accumulation of microtubules in cardiomyocytes is a feature of pressure overload hypertrophy that may impair contractile function and contribute to heart failure (15, 41). Aberrant stabilization and accumulation of MTs are believed to contribute to contractile dysfunction, in part, by increasing the viscous load on the myofilaments (36, 41). Microtubules also play an important role in ion channel trafficking (29), mRNA transport (22, 27), and intracellular organization (38, 40, 43), and disruption of these microtubule functions by aberrant stabilization in pressure overload may also contribute to adverse remodeling and heart failure. In support of a role for MT stabilization in contractile dysfunction, we found that chronic (every other day) treatment with colchicine to reduce MT densification attenuated hypertrophy and improved contractile function in Balb-c mice exposed to pressure overload produced by transverse aortic constriction (TAC) (7). Identification of endogenous pathways that regulate cardiac microtubule dynamics may thus provide pharmacological targets for attenuating microtubule-related ventricular dysfunction in the hypertrophied heart.

AMPK is a metabolic sensor that limits anabolic processes when ATP production is insufficient to meet the energetic demands of the cell. Activated by increased AMP/ATP ratio, AMPK reduces energy-consuming processes, such as protein synthesis by inhibiting the mTOR signaling pathway (10, 13, 28). In the heart, deletion of the AMPK α_2 catalytic subunit exacerbates cardiac hypertrophy and heart failure in response to pressure overload, and this is associated with up-regulated mTOR signaling (46). While mTOR signaling is important for the hypertrophic response (31), recent evidence suggests mTOR also has antiapoptotic, anti-inflammatory, and metabolic functions that may be important for adaptive hypertrophy (30, 33, 45). Thus, while increased mTOR signaling may contribute to cardiac hypertrophy, it is not sufficient to explain the worsened cardiac function in AMPK α_2 KO hearts after TAC.

In addition to regulating protein synthesis, AMPK can regulate the cytoskeleton. AMPK is a member of the MARK/PAR kinase subfamily (microtubule affinity-regulating kinase), which phosphorylates MAPs (microtubule-associated proteins) to influence MT dynamics and cell polarity (18). AMPK was recently shown to directly phosphorylate the microtubule-associated protein tau and alter its binding to neuronal microtubules (39). In addition, AMPK phosphorylation of the plus end tracking protein clip-170 increased MT polymerization speed and was found necessary for directed cell migration, while reduced CLIP-170 phosphorylation by AMPK resulted in less dynamic MTs (20). Whether AMPK regulates MT dynamics in cardiomyocytes, however, and how this influences the hypertrophic response is not known.

Here, we investigated AMPK regulation of cardiac microtubules during hypertrophy of cultured rat neonatal cardiomyocytes and in pressure overload-induced cardiac hypertrophy using the TAC model in mice. Our data identify, for the first time, the cardiac microtubule cytoskeleton as a sensitive target of AMPK activity, and suggest a novel role for AMPK in limiting accumulation and densification of microtubules in response to pressure overload.

Address for reprint requests and other correspondence: R. Bache, Univ. of Minnesota, Mayo Mail Code 508, 401 Delaware St. SE, Minneapolis, MN 55455 (e-mail: bache001@umn.edu).

 0363-6135/13 Copyright © 2013 the American Physiological Society

Downloaded from http://ajpheart.physiology.org/ at Univ of Minnesota on July 5, 2013

MATERIALS AND METHODS

Animals and TAC. Animals were housed in an air-conditioned room with a 12:12-h light-dark cycle, received standard rodent chow, and drank tap water. AMPK α_2 KO and control wild-type mice were generated and used for sham or subjected to TAC, as previously described (11). This study was approved by the Institutional Animal Care and Use Committee of University of Minnesota.

Echocardiography. Mice were anesthetized with 1.5% isoflurane, and echocardiographic images were obtained with a Visualsonics high-resolution Veve 660 system, as previously described (46). Left ventricular (LV) diameter, shortening fraction, and wall thickness were measured from two-dimensional guided short-axis M-Mode views of the LV.

Neonatal rat ventricular cardiomyocyte culture and protein analysis. Neonatal rat ventricular cardiomyocytes (NRVMs) were isolated from 2-day-old Sprague-Dawley rats by enzymatic digestion and separated from nonmuscle cells on a discontinuous Percoll gradient. NRVMs were isolated from 2-day-old Sprague-Dawley rats by enzymatic digestion and separated from nonmuscle cells on a discontinuous Percoll gradient, as previously described (7). A total of 2–4 million viable myocytes were isolated per ventricle with very little fibroblast contamination (<2%) and plated on gelatin-coated plates in serum-containing DMEM (0.5–1 × 10^5 cells/cm^2) and incubated for 48 h to allow attachment and spreading, after which, the medium was replaced with serum-free media for 24 h prior to treatment. Regarding adenovirus infection, one day after plating, cardiomyocytes were infected with the indicated multiplicity of infection of constitutively active AMPK (caAMPK), WT AMPK (8), or β-Gal-expressing adenovirus. After 8 h to allow infection, media were changed, and cells were allowed to grow for an additional 16 h in serum before replacement with serum-free media and treatments.

Knockdown of microtubule-associated protein. For RNAi treatment, cells were plated at 1 × 10^5 cells/cm^2. After 24 h, cells were transfected overnight using Lipofectamine 2000 (Invitrogen) with 10 pm/well rat microtubule-associated protein 4 (MAP4) siRNA or nontargeting control siRNA (Dharmacon, Lafayette, CO) in the continued presence of 10% FCS. The next day, the media were replaced with DMEM containing insulin/transferrin/selenium solution (Sigma) and incubated for an additional 24–48 h before replacement with growth factor-free media (DMEM) for 24 h before treatment with phenylephrine (PE) or 5-aminoimidazole carboxamide ribonucleotide (AICAR).

Isolation of cytoskeletal fractions from NRVMs. Cell lysates were collected in Triton lysis buffer and separated into soluble (cytosol and membrane) and insoluble (cytoskeletal) fractions, as previously described (7) for Western blot analysis. Regarding immunofluorescence staining of microtubules, NRVMs were fixed in ice-cold methanol for 10 min, permeabilized with 0.2% Triton/PBS, blocked 30 min at RT with 2% FCS/PBS, and incubated with primary antibodies overnight. Primary antibodies were detected using Alexa Fluor 488 or Alexa Fluor 555 secondary antibodies (Molecular Probes, Carlsbad, CA). For cell area measurement, cells were plated at 0.5 × 10^5 cells/cm^2. After treatment, cells were fixed with 10% methanol as for immunofluorescence and then stained using 0.3% Coomassie blue for 5 min, followed by three rinses in PBS and photographed under phase contrast. Cell area was analyzed using Image J 1.34s (U.S. National Institutes of Health, Bethesda, MD). At least 100 individual cells were measured per experiment.

Leucine incorporation by cardiomyocytes. One microcurie of [3-H]leucine (Perkin Elmer, Waltham, MA) was added per well in 24-well plates. After 48 h of treatment, cells were washed once in PBS, fixed 10 min in 10% TCA, washed again in TCA, dissolved in 5% SDS, 0.1 M NaOH, and [^{3}H] incorporation was measured in a liquid scintillation counter.

Isolation of microtubules and other fractions from mouse cardiac tissue. Mouse hearts were processed for separation of free and polymerized tubulin, as previously described (41), with minor modifications. Mouse hearts were snap frozen at the time of collection, then pulverized under liquid nitrogen. Approximately one-half of the pulverized tissue was homogenized in 1 ml of MT stabilization buffer at 20,000 rpms for 20 s and processed for separation of free and polymerized tubulin, as previously described (41). The other half of frozen powdered tissue was homogenized in 1% Triton X-100, 1 mM EDTA, 100 mM NaCl, 1 mM vanadate, 1 mM PMSF, 1 × protease inhibitor cocktail (Boehringer Mannheim, Mannheim, Germany), and 10% glycerol, in 10 mM Tris·HCl at pH 7.4. Lysates were centrifuged at 14,000 *g*, and Triton-soluble supernate was separated from the Triton-insoluble pellet. The pellet was washed again in lysis buffer, and then resuspended in 2 × SDS loading buffer containing phosphatase and protease inhibitors and 2.5% β-mercaptoethanol and boiled. For protein concentrations, lysates were precipitated using 10% TCA, and measured using the BCA assay (Pierce Biotechnology, Rockford, IL).

Phosphorylation of MAP4 peptide by purified active AMPK and mass spectrometry analysis. Five micrograms of MAP4 peptide PDLKNVRSKVGSTENIKHQPGGG, corresponding to amino acids 903–925 of mouse MAP4, was incubated in 5 mM MOPS pH 7.2, 2.5 mM β-glycerophosphate, 1 mM EGTA, 0.4 mM EDTA, 5 mM $MgCl_2$, 0.5 mM DTT, 50 μM ATP, 0.1 mM AMP, with 100-ng purified active AMPK $\alpha_2\beta_2\gamma_2$ (SignalChem, Richmond, BC, Canada) for 1 h at 23°C. Peptide alone or peptide + active enzyme samples were analyzed by LCMS at the Center for Mass Spectrometry and Proteomics at the University of Minnesota using an LTQ Orbitrap Velos mass spectrometer. Data were analyzed using Xcalibur software (Thermo Scientific, Waltham, MA).

Chemicals and antibodies. Phenylephrine and AICAR were from Sigma (St. Louis, MO). Antibodies against α-tubulin (Cell Signaling, Danvers, MA), MAP4, and detyrosinated tubulin (Glu-tubulin) (Millipore, Temecula, CA), Phospho-MAP4^{Ser768} (this is Ser-941 of "full-length" human MAP4) (Biolegend, San Diego, CA) sarcomeric actin, β-actin, α-tubulin, β-tubulin, (Sigma) desmin, and anti-cardiac MHC (Abcam, Cambridge, MA) were used for detection of proteins in Western blot analysis and/or immunofluorescence.

Data and statistical analyses. All values were expressed as means ± SE. Statistical significance was defined as $P < 0.05$. One-way ANOVA was used to test each variable for differences among the treatment groups with StatView (SAS Institute, Cary, NC). If the ANOVA demonstrated a significant effect, post hoc pairwise comparisons were made with the Student's *t*-test.

RESULTS

AMPK regulation of cell size, protein synthesis, and microtubule dynamics. Cell enlargement requires increased protein synthesis, as well as coordinated expansion of the cytoskeletal networks that support the new cell dimensions. While investigating the role of AMPK in cardiomyocyte hypertrophy, we observed that AICAR treatment was able to limit cell area at a low dose (0.1 mM) that did not block protein synthesis (Fig. 1*D–F*). This suggests an AICAR effect on cell spreading or cell shape that may occur independently of the well-known AMPK inhibitory effects on protein synthesis. Because cell spreading is mediated by changes in cytoskeletal dynamics, we examined expression and partitioning (cytosolic vs. cytoskeletal) of cardiomyocyte cytoskeleton proteins in response to PE stimulation and whether this is altered by AICAR treatment. Gentle lysis of cardiomyocytes into Triton-soluble (membrane and cytosol; sol) and insoluble (cytoskeleton and nucleus; csk) fractions revealed that activation of AMPK with AICAR had little effect on

Downloaded from http://ajpheart.physiology.org/ at Univ of Minnesota on July 5, 2013

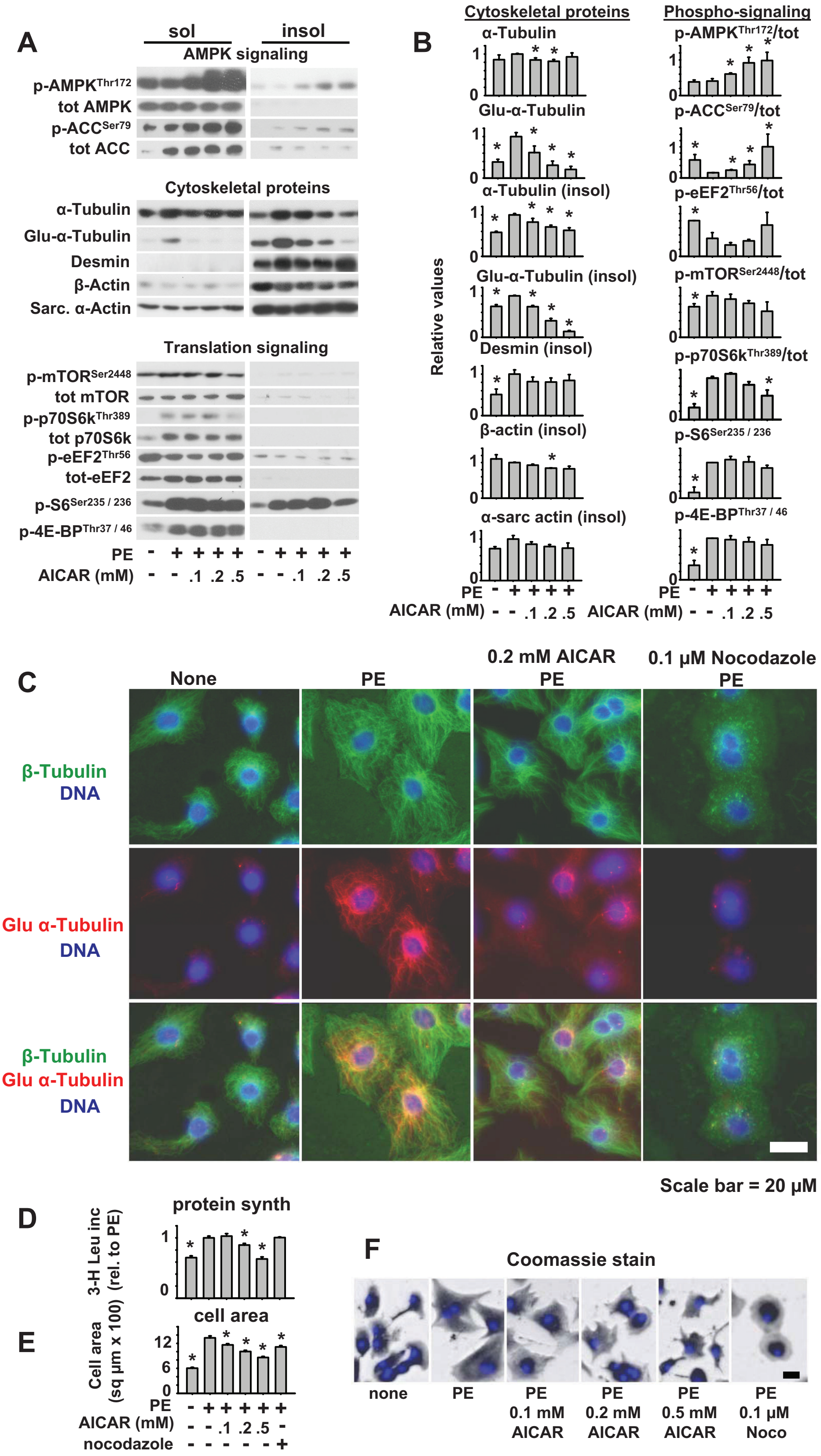

Fig. 1. AMPK regulation of microtubule (MT) stability, cell size, and protein synthesis in neonatal rat cardiomyocytes. Neonatal rat cardiomyocytes were treated with phenylephrine (50 μM) and increasing doses of 5-aminoimidazole carboxamide ribonucleotide (AICAR). Triton-soluble and insoluble fractions were collected after 24 h of treatment, and the expression and partitioning of the indicated proteins were analyzed by Western blot analysis (*A*) and quantified (*B*). Immunofluorescent staining of β-tubulin (green) and detyrosinated tubulin (Glu-tubulin; red) and DNA (Hoescht; blue) were examined at 24 h of treatment (*C*). For [3-H]leucine incorporation (Leu inc; *D*) and cell area (*E*), 0.1 μM nocodazole was also included to determine effects of MT disruption. For measuring cell area, fixed cells were stained with Coomassie blue (*F*). Graphs represent protein levels relative to PE-treated cardiomyocytes averaged from three to six experiments $*P < 0.05$ compared with PE-treated cells.

Downloaded from http://ajpheart.physiology.org/ at Univ of Minnesota on July 5, 2013

PE-induced assembly of myofilaments (csk-sarcomeric actin or β-actin) or intermediate filaments (csk-desmin), while it dose-dependently reduced microtubule (csk-tubulin) levels (Fig. 1, *A–C*). Examination of stabilized MTs using antibodies to detyrosinated tubulin (the c-terminus tyrosine is cleaved in longer-lived polymerized microtubules, leaving a c-terminal glutamine, Glu-tubulin) confirmed that microtubule longevity was much reduced in the presence of AICAR (Fig. 1, *A–C*). Interestingly, MT stability was more sensitive to AMPK activation than inhibition of mTOR, EEF2, or protein synthesis, as AICAR attenuated microtubule stability and cell area (Fig. 1, *A*, *B*, and *E* and *F*) at doses (0.1 mM) that did not block mTORC1 signaling, EEF2 phosphorylation (Fig. 1, *A* and *B*), or protein synthesis (Fig. 1*D*). Similar to low-dose AICAR treatment, nocodazol inhibition of microtubule growth reduced cell area without inhibiting protein synthesis (Fig. 1, *D* and *E*), indicating that blocking microtubule proliferation can attenuate cell spreading, independent of overall changes in protein synthesis. One explanation for the decreased cell area despite equal levels of protein synthesis is that MT-dependent distribution of proteins or organelles to the cell periphery requires more persistent elongated microtubule tracks, which were diminished in the presence of AICAR. This possibility is supported by Coomassie staining of cellular protein distribution in fixed cells (Fig. 1*F*), which shows proteins more highly concentrated in perinuclear regions of cells treated with AICAR or nocodazole, while cells treated with PE alone have more evenly distributed proteins that reach the cell periphery.

To rule out the possibility that reduction in MT stability is an indirect effect of less hypertrophy, cardiomyocytes were treated with phenylephrine for 72 h to establish hypertrophy and microtubule densification, then treated briefly (3 h) with AICAR. Three hours of AICAR treatment reduced Glu-tubulin levels (Fig. 2, *A–C*), but it did not significantly reduce overall cytoskeletal tubulin, indicating that AMPK reduction of microtubule stability can occur independent of AMPK effects on hypertrophy, or on overall microtubule levels.

To determine whether the reduction of MTs is due to activated AMPK rather than an indirect effect of AICAR, cells were infected with adenovirus expressing constitutively active AMPK (CA-AMPK) for 24 h, then treated with phenylephrine for an additional 48 h. Similar to AICAR treatment, CA-AMPK reduced microtubule density and Glu-tubulin levels in phenylephrine-treated cells as demonstrated by immunofluorescence staining (Fig. 2*D*). Similar to low-dose AICAR treatment, CA-AMPK reduced cytoskeletal tubulin levels (Fig. 2*E*) and cell area (Fig. 2*F*) without inhibiting protein synthesis (Fig. 2*G*). These data indicate that AMPK regulates MT dynamics, which can influence cell spreading independent of protein synthesis.

To further examine the role of AMPK in microtubule regulation, we overexpressed wild-type AMPK-α_2 in neonatal cardiomyocytes and examined whether this influences phenylephrine-induced MT stabilization and accumulation. Adenoviral overexpression of WT AMPK resulted in spontaneously increased AMPK^{Thr172} phosphorylation, a reduction in stabilized microtubules (detyrosinated tubulin), and a reduction in tubulin association with the Triton-insoluble cytoskeleton, similar to the effects of activating AMPK using AICAR (Fig. 2, *H* and *I*), These data are further evidence that AMPK regulates microtubule stability in cardiomyocytes.

The finding that MTs are a sensitive target of AMPK in neonatal cardiomyocytes led us to investigate whether AMPK may influence cardiac MT dynamics in vivo, where microtubule densification is believed to contribute to pressure overload-induced heart failure.

AMPK regulation of cardiac MT dynamics in pressure overload hypertrophy. We have previously demonstrated that TAC-induced hypertrophy, LV dysfunction, and heart failure are exacerbated by AMPK α_2 KO (46). Because we observed that AMPK activation can reduce MT stabilization in neonatal cardiomyocytes exposed to hypertrophic signals, we examined the influence of AMPK α_2 (AMPK α_2 KO), the predominant AMPK catalytic subunit in the heart (34), on cardiac microtubule dynamics in mice under basal conditions or hypertrophic stress (transverse aortic constriction, TAC) (Fig. 3, *A* and *B*). Interestingly AMPK α_2 KO mice had ~60% increase in polymerized tubulin levels compared with WT mice under basal conditions. Three weeks of TAC resulted in further increases in free and polymerized tubulin levels in both WT and AMPK α_2 KO mice, but the increase in polymerized and free tubulin was significantly greater in the AMPK α_2 KO mice. Detyrosinated tubulin (Glu-tubulin) levels were also significantly higher in AMPK α_2 KO mice exposed to TAC, indicating an increase in microtubule stability. Interestingly, in AMPK KO mice, the levels of polymerized tubulin exhibited a striking correlation with increased lung to body weight ratio (indicative of pulmonary congestion) and reduced ejection fraction (indicative of contractile dysfunction) (Fig. 3, *C* and *D*). Taken together, these results suggest AMPK α_2 plays a role in limiting pressure overload-induced accumulation and stabilization of cardiac microtubules, and they identify an association between increased MT accumulation and left ventricular dysfunction.

AMPK regulation of microtubule-associated protein 4. To investigate mechanism(s) of AMPK regulation of cardiac MTs,

Fig. 2. Brief AICAR treatment or constitutive AMPK activity reduce MT stability. Neonatal cardiomyocytes were stimulated to hypertrophy with phenylephrine for 72 h, and then treated for 3 h with AICAR (0.2 mM). Triton-soluble and insoluble tubulin and Glu tubulin levels were analyzed by Western blot (*A*) and quantified (*B*). Microtubules were also analyzed by immunofluorescence (*C*), detecting β-tubulin and Glu-α-tubulin with Alexa Fluor 488 (green) and Alexa Fluor 555 (red), respectively. DNA was detected using Hoescht stain (blue). Constitutively active AMPK reduces MT stability. Neonatal cardiomyocytes were treated with 5 multiplicity of infection (MOI) control adenovirus (β-gal) or CA-AMPK adenovirus for 24 h, prior to 48 h phenylephrine treatment, and analyzed by immunofluorescence staining of α-tubulin (green), Glu- α-tubulin (red), Hoescht staining of DNA (blue) (*D*); scale bar = 25 μM. Western blot of cytoskeletal tubulin (*E*), cell area measurement (*F*), and [3-H]leucine incorporation (*G*). Overexpression of wild-type (WT) AMPKα_2 reduces microtubule stability. Neonatal cardiomyocytes were treated with control adenovirus (β-gal) or myc-tagged WT-AMPK-expressing adenovirus at an MOI of 30 for 24 h, prior to 48 h phenylephrine treatment. Additionally, AICAR was added to β-gal-treated cells in the final 24 h as a control for AMPK activation. Triton-soluble (sol) and triton-insoluble (csk) fractions were examined by Western blot for AMPK expression and phosphorylation, as well as levels of total and detyrosinated tubulin. Sarcomeric α-actin was used as a loading control. Graphs represent averages from at least three experiments. *$P < 0.05$ compared with PE-treated cells.

Downloaded from http://ajpheart.physiology.org/ at Univ of Minnesota on July 5, 2013

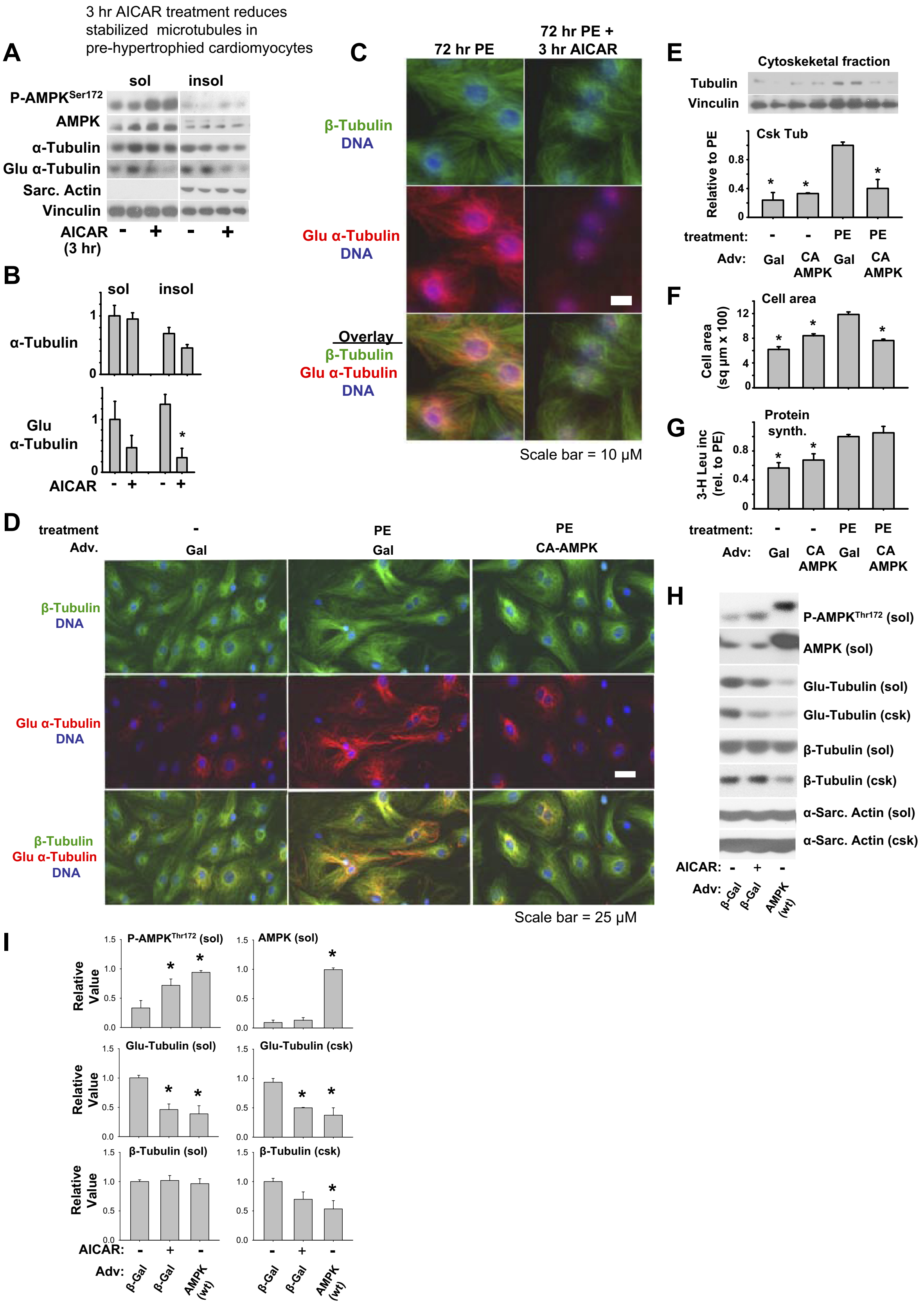

Downloaded from http://ajpheart.physiology.org/ at Univ of Minnesota on July 5, 2013

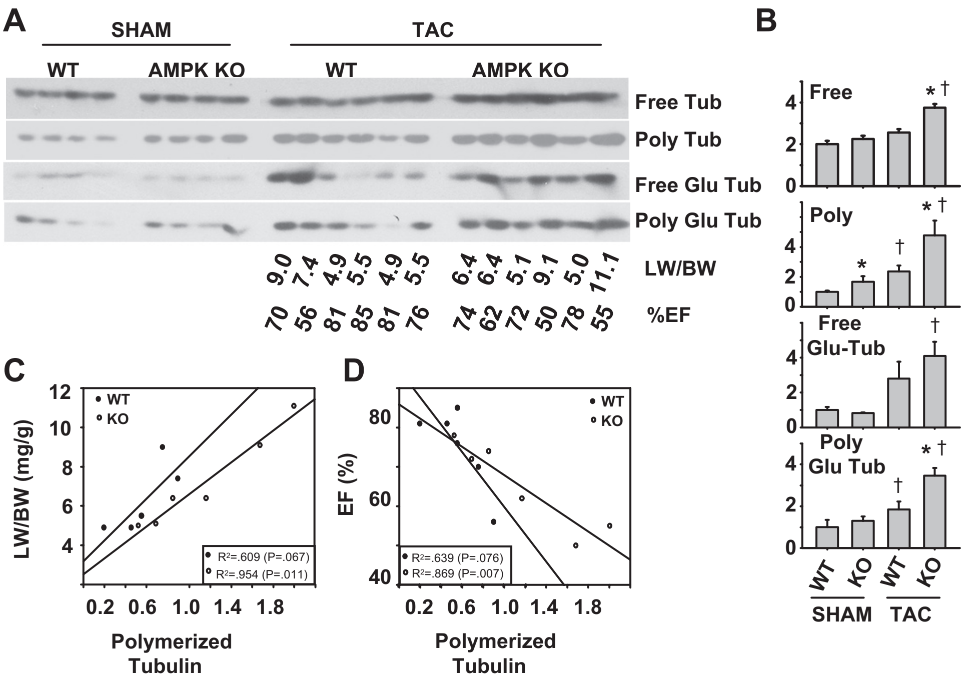

Fig. 3. In response to pressure overload, AMPK α_2 knockout (KO) mice show an exacerbated microtubule stabilization and accumulation, which correlates with contractile dysfunction. Free and polymerized MT fractions were isolated from WT and AMPK α_2 KO mice after 3 wk of transverse aortic constriction (TAC) and analyzed by Western blot for α-tubulin or Glu- α-tubulin (Fig. 5, *A* and *B*). Lung weight to body weight ratio (LW/BW) (*C*) or % left ventricular ejection fraction (EF; *D*) of WT (●) and AMPK KO mice (○) exposed to TAC were plotted vs. polymerized tubulin levels from the corresponding hearts. **P* < 0.05 comparing WT to KO under same conditions. †*P* < 0.05 comparing sham to TAC.

we examined MAP4. MAP4 binds to and stabilizes microtubules in response to pressure overload, but its affinity for microtubules is reduced by phosphorylation at Ser-914 (ms) [equivalent to Ser-915 (rat), Ser-924 (cat), and Ser-941(hu) (4, 6, 12)]. To determine whether MAP4 expression or phosphorylation is regulated by AMPK α_2 in vivo, we analyzed the Triton-soluble and insoluble ventricular lysates of WT and AMPK α_2 KO mice exposed to sham or TAC conditions. In Triton X-100 tissue homogenates, mechanical homogenization in the presence of detergent, phosphatase inhibitors, and low temperature causes most of the tubulin to fractionate with the Triton-soluble fraction. Interestingly, most of the MAP4 protein partitioned in the Triton-insoluble fraction (not shown). This suggests that MAP4 also associates with other more stable or Triton-insoluble cytoskeletal elements of the cell in addition to microtubules, consistent with a recent report demonstrating MAP4 also binds to actin (19). Under basal conditions, insoluble MAP4 levels were significantly higher in AMPK α_2 KO hearts than WT, while phosphorylation of insoluble MAP4 relative to total MAP4 was reduced in AMPK α_2 KO samples (Fig. 4, *A* and *B*). These differences in expression level or phosphorylation were not observed after TAC, however, whereas MAP4 expression increased in both WT and AMPK α_2 KO (albeit with a higher relative increase in WT hearts compared with the low levels under sham conditions). In response to TAC, phosphorylation of Triton-insoluble MAP4^{Ser914} was reduced as previously observed in cats (4), with no significant difference between WT and KO. In the Triton-soluble fraction, however, MAP4^{Ser914} phosphorylation was increased in WT samples, while the increase in MAP4^{Ser914} phosphorylation was significantly attenuated in AMPK α_2 KO, consistent with a role for AMPK α_2 in promoting MAP4 phosphorylation and reducing MT stability.

To determine whether activation of AMPK can increase MAP4 phosphorylation, we treated prehypertrophied (after 48 h of phenylephrine exposure) neonatal cardiomyocytes for an additional 24 h with phenylephrine in the absence or presence of 0.2 mM AICAR. AICAR exposure resulted in increased MAP4 phosphorylation and a trend toward reduced total MAP4 levels. At the same time, stabilized MTs, but not total MTs, in the same fraction were dramatically reduced, consistent with a role for MAP4 in MT stabilization. (Fig. 5, *A* and *B*). Recently, AMPK was found to phosphorylate serine-262 within the KXGS motif in the microtubule-associated protein tau (39). Ser-262 in human tau is analogous to Ser-914 in the KXGS motif in mouse MAP4 (87% homology in the 22 amino acids surrounding Ser-914). To determine whether AMPK can directly phosphorylate Ser-914 of MAP4 in vitro, we incubated active WT, AMPK $\alpha_2/\beta_2/\gamma_2$ with a peptide corresponding to amino acids 903 to 925 in mouse MAP4 and used LCMS to detect phosphorylation of the peptide. LCMS analysis showed that this peptide was monophosphorylated by AMPK on serine-914 within the KXGS motif (data not shown), suggesting AMPK can directly phosphorylate MAP4 at serine-914.

To verify the role of MAP4 in cardiomyocyte microtubule stability, we used RNAi to reduce MAP4 expression. MAP4 RNAi treatment of neonatal cardiomyocytes reduced stabilized MT levels (Glu-tubulin), compared with control nontargeting RNAi (Fig. 5, *C* and *D*). Interestingly, MAP4 depletion also altered MT organization, so that microtubules were sparsely distributed in some areas of the cell, while densely aligned along the cell periphery in others. Some cells treated with MAP4 RNAi also exhibited abnormal nuclear positioning, so that nuclei were observed along the cell periphery rather than the center (Fig. 5*C*). MAP4 also has been shown to bind to and regulate actin filaments (19), so MAP4 may influence cytoskeletal organization and nuclear positioning through effects on actin and/or microtubules. Interestingly, AICAR treatment still further reduced stabilized MTs in MAP4-deficient cells (Fig. 5*D*), suggesting AMPK activation may also reduce MT stabilization through mechanisms that are independent of MAP4.

Downloaded from http://ajpheart.physiology.org/ at Univ of Minnesota on July 5, 2013

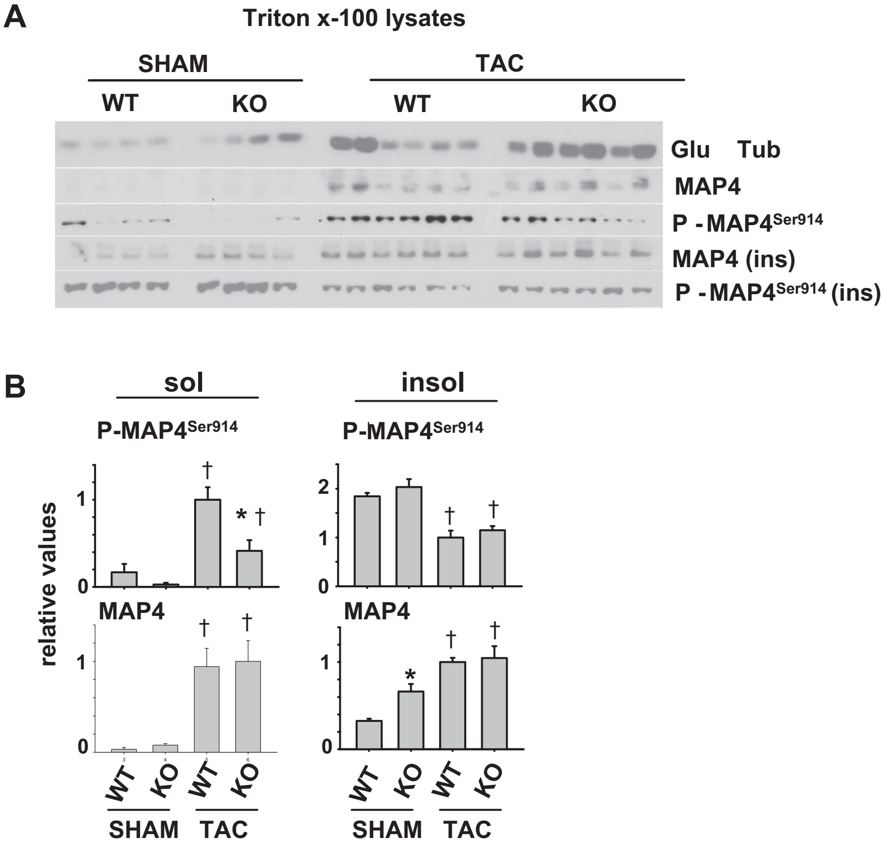

Fig. 4. AMPK α_2 KO reduces phosphorylation of microtubule-associated protein 4^{Ser914} ($MAP4^{Ser914}$). Triton-soluble and insoluble lysates from WT and AMPK α_2 KO mice exposed to sham or TAC were analyzed by Western blot (*A*) and quantified (*B*) for the indicated proteins. *$P < 0.05$ comparing WT to KO under same conditions. †$P < 0.05$ comparing sham to TAC.

Downloaded from http://ajpheart.physiology.org/ at Univ of Minnesota on July 5, 2013

DISCUSSION

AMPK has long been recognized as a metabolic sensor that reduces energy-consuming processes (such as protein synthesis), while increasing energy production (glucose transport, fatty acid oxidation, mitochondria biogenesis). Our data suggest a novel role for cardiac AMPK in attenuating the stabilization and densification of MTs that occurs in response to hypertrophic stress. AMPK prevention of microtubule proliferation can limit expansion of cell area in cultured cells, independent of previously identified AMPK inhibitory effects on protein synthesis. More importantly, our data suggest AMPK limits microtubule stabilization that occurs in response to pressure overload, where microtubule proliferation is believed to contribute to heart failure.

Microtubules and heart failure. There is evidence that microtubule densification contributes to contractile dysfunction through mechanical impairment of contractility (15, 36, 41). Reducing the viscous load upon myofilaments imposed by microtubules in the heart may allow AMPK to improve contractile function without additional energy expenditure. However, because microtubules play an important role in intracellular transport and subcellular organization, altered microtubule dynamics in pressure overload could prove maladaptive through other mechanisms in addition to increasing myofilament load. For instance, microtubule decoration by MAP4 (which plays a role in pressure overload-induced MT stabilization) can inhibit transport of receptors (2) and mRNAs (25, 26). Disruption of mRNA and protein transport may impair compensatory hypertrophy and promote the transition to decompensation, which is associated with increased MT accumulation in pressure overload-induced heart failure (35). Microtubules also play an important role in organelle transport and distribution (1, 9, 38, 40, 44), and decoration of microtubules by MAPs may, thus, alter intracellular organization. Interestingly, microtubules were recently shown to mediate stretch-induced NADPH oxidase activation, oxidative stress, and calcium sparks (14, 23), suggesting a potential role for increased MTs in oxidative stress and altered calcium dynamics observed in heart failure. In support of a role for MT accumulation in contractile dysfunction in AMPK KO mice, we observed that polymerized microtubule levels correlated strikingly with reduced left ventricular ejection fraction and pulmonary congestion in mice exposed to TAC. Furthermore, our previous study demonstrated that periodic colchicine treatment attenuated chamber dilation and improved contractile function in Balb-c mice exposed to pressure overload (7). The present data suggest that agents that activate AMPK may similarly preserve contractile function by preventing MT densification.

How does AMPK regulate microtubules? Recently, a mechanism for MT stabilization during pressure overload hypertrophy was identified. MAP4, which is upregulated in response to pressure overload (24), binds to and promotes MT stabilization (12, 37). However, when phosphorylated at Ser-924 of the KXGS motif (equivalent to Ser-914 in mice) within the MT binding domain, this interaction is inhibited and MT stability is reduced (4). Cheng et al. (3) showed that increased phospha-

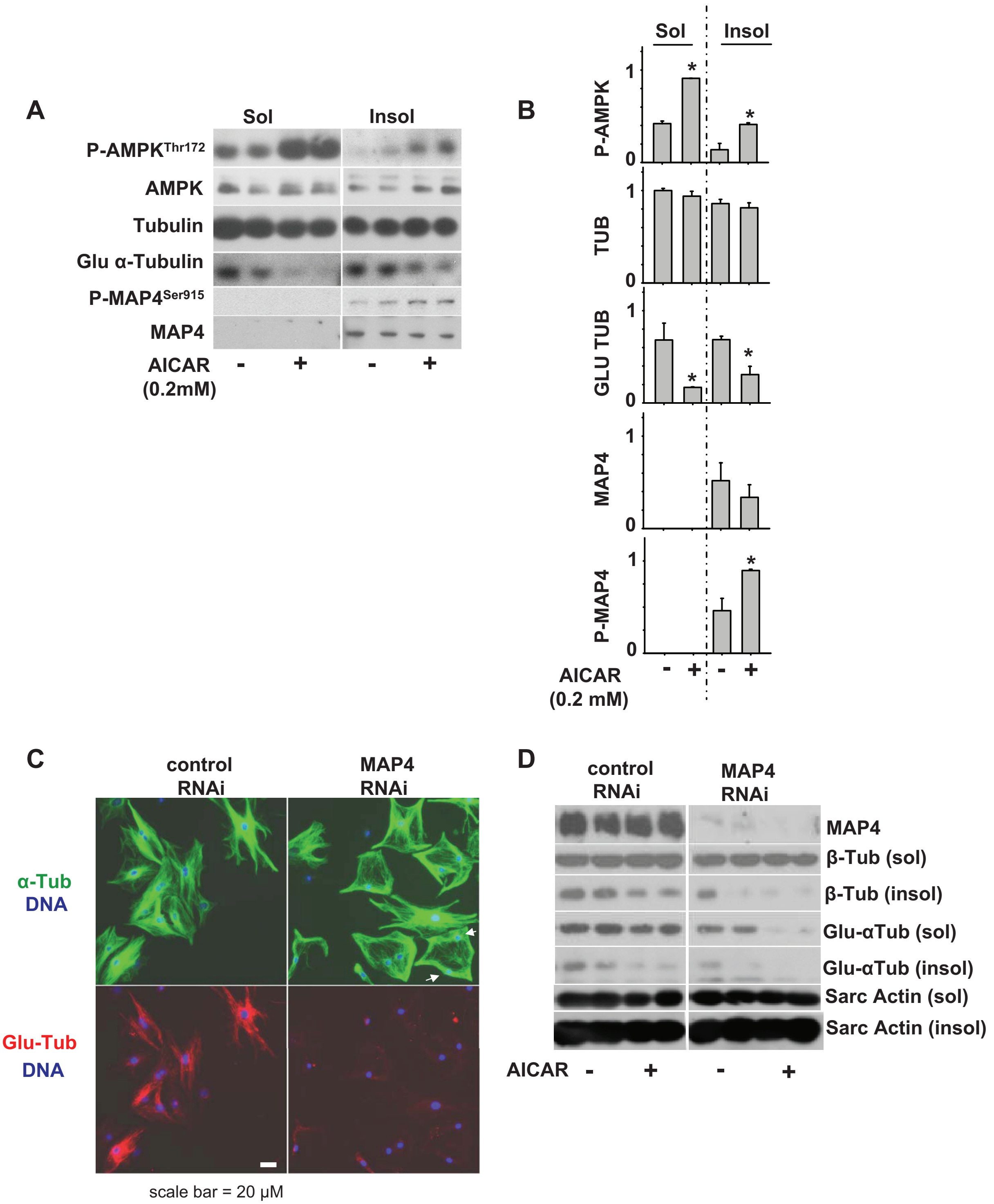

Fig. 5. AMPK activation increases phosphorylation of MAP4. Prehypertrophied neonatal cardiomyocytes (48-h exposure to 50 μM phenylephrine) were exposed to an additional 24 h of phenylephrine treatment in the presence or absence of 0.2 mM AICAR, prior to analysis by Western blot analysis (*A*) and quantification (*B*) of phosphor-AMPK (P-AMPK), tubulin, Glu-tubulin, MAP4, and phosphor-MAP4^{Ser915} (P-MAP4) (Note Ser-915 in rat MAP4 is equivalent to Ser-914 in mouse MAP4). MAP4 is important for cardiomyocyte microtubule stability and organization. *C*: cardiomyocytes were exposed to MAP4 RNAi or nontargeting control RNAi for 72 h and analyzed by immunofluorescence (*C*). Arrows point to abnormally localized nuclei in MAP4 RNAi-treated cells. *D*: microtubule stability was also examined by Western blot in control and MAP4 RNAi-treated cells after AICAR treatment (0.2 mM) for 24 h. *$P < 0.05$ compared with phenylephrine-treated cells.

Downloaded from http://ajpheart.physiology.org/ at Univ of Minnesota on July 5, 2013

tase activity during pressure overload results in dephosphorylation of MAP4^{Ser924}, thereby promoting MT assembly and stabilization. We find that activation of AMPK increases MAP4 phosphorylation at the analogous site in rat cardiomyocytes. Conversely, MAP4 phosphorylation at this site is reduced in AMPK α_2 KO mice. Thus, it appears that AMPK plays a role in counteracting the effects of PP2A on MT stability by reducing MAP4 affinity for MTs.

AMPK is a member of the MARK/PAR kinase subfamily of Ser/Thr kinases, some of which have been shown to directly phosphorylate MAP4 on KXGS motifs to reduce affinity for microtubules (5, 12). The MAP4 kinases in the heart have not been identified. It was recently demonstrated that AMPK can directly phosphorylate Ser-262 of the KXGS motif in the microtubule binding domain of tau, the neuronal MAP associated with Alzheimer's disease (39). The 22 amino acids flanking Ser-914 in MAP4 and Ser-262 in tau are ~87% homologous (19/22). In support of direct phosphorylation of MAP4 by AMPK, we find that purified active AMPK can phosphorylate this same site in vitro. Because MAP4 phosphorylation was not completely removed in AMPK KO mice, it is likely that other kinases also regulate MAP4 phosphorylation in the heart. This could be due to AMPK α_1 activity, or the activity of an AMPK-related kinase, such as MARK2. MARK2 exhibits reduced activity in response to right ventricular pressure overload in cats at the same time that MAP4 phosphorylation is reduced (3). However, in this mouse model, we did not observe reduced MARK2 activation (as indicated by phosphorylation at Ser-215 in the activation loop; not shown) in response to TAC. Although our results suggest a role for AMPK in cardiac MAP4 phosphorylation, it will be important to determine the role of other MARKs and AMPK-related kinases in cardiac MAP4 phosphorylation, as well as the counterpoised MAP4 phosphatases (PP2A), to acquire a more complete understanding of the signaling pathways that regulate MAP4 and cardiac MT dynamics.

In addition to phosphorylation of MAP4, AMPK may influence MT dynamics through MAP4-independent mechanisms. This is suggested by the reduction in MT stability by AICAR, even when MAP4 was eliminated using RNAi. AMPK has also been shown to regulate microtubule dynamics through phosphorylation of clip 170 (21). However, RNAi depletion of clip 170 did not result in obvious effects on MT stability (not shown). We did observe a slight reduction in free tubulin in cells treated with AICAR, while total tubulin was higher in AMPK KO mice after TAC. Persistently high levels of tubulin synthesis are necessary for MT growth and stability, so it is possible that AMPK reduction of tubulin heterodimer synthesis may also contribute to reduced MT levels. However, we still observe rapid MT destabilization in prehypertrophied cells, even as free or polymerized tubulin levels change only slightly, so other AMPK-dependent mechanisms of MT destabilization are also likely to be involved. While our data identify MAP4 phosphorylation as one mechanism by which AMPK activity may promote MT destabilization, additional experiments will be necessary to identify the MAP4-independent mechanisms of cardiac MT regulation by AMPK.

In summary, we have identified a new role for AMPK in limiting stabilization and accumulation of microtubules during pressure overload-induced cardiac hypertrophy. Future studies will be designed to determine whether treatments that activate cardiac AMPK can prevent microtubule accumulation during pressure overload, and the extent to which AMPK regulation of microtubule dynamics contributes to the cardioprotective effects of AMPK.

ACKNOWLEDGMENTS

John Fassett is a recipient of a Scientist Development Award from the American Heart Association.

GRANTS

This study was supported by U.S. Public Health Service Grants HL21872 and HL71790 from the National Heart, Lung and Blood Institute, and Research Grants 0330136N and 0160275Z from the American Heart Association.

DISCLOSURES

No conflicts of interest, financial or otherwise, are declared by the authors.

AUTHOR CONTRIBUTIONS

Author contributions: J.T.F. and Y.C. conception and design of research; J.T.F., X.H., X.X., Z.L., P.Z., and Y.C. performed experiments; J.T.F., X.X., and Y.C. analyzed data; J.T.F., Y.C., and R.J.B. interpreted results of experiments; J.T.F. prepared figures; J.T.F. drafted manuscript; J.T.F., Y.C., and R.J.B. edited and revised manuscript; J.T.F., X.H., X.X., Z.L., P.Z., Y.C., and R.J.B. approved final version of manuscript.

REFERENCES

1. **Bannai H, Inoue T, Nakayama T, Hattori M, Mikoshiba K.** Kinesin dependent, rapid, bi-directional transport of ER sub-compartment in dendrites of hippocampal neurons. *J Cell Sci* 117: 163–175, 2004.
2. **Cheng G, Qiao F, Gallien TN, Kuppuswamy D, Cooper G.** Inhibition of β-adrenergic receptor trafficking in adult cardiocytes by MAP4 decoration of microtubules. *Am J Physiol Heart Circ Physiol* 288: H1193–H1202, 2005.
3. **Cheng G, Takahashi M, Shunmugavel A, Wallenborn JG, DePaoli-Roach AA, Gergs U, Neumann J, Kuppuswamy D, Menick DR, Cooper G.** Basis for MAP4 dephosphorylation-related microtubule network densification in pressure overload cardiac hypertrophy. *J Biol Chem* 285: 38125–38140, 2010.
4. **Chinnakkannu P, Samanna V, Cheng G, Ablonczy Z, Baicu CF, Bethard JR, Menick DR, Kuppuswamy D, Cooper G.** Site-specific microtubule-associated protein 4 dephosphorylation causes microtubule network densification in pressure overload cardiac hypertrophy. *J Biol Chem* 285: 21837–21848, 2010.
5. **Drewes G, Ebneth A, Preuss U, Mandelkow EM, Mandelkow E.** MARK, a novel family of protein kinases that phosphorylate microtubule-associated proteins and trigger microtubule disruption. *Cell* 89: 297–308, 1997.
6. **Ebneth A, Drewes G, Mandelkow EM, Mandelkow E.** Phosphorylation of MAP2c and MAP4 by MARK kinases leads to the destabilization of microtubules in cells. *Cell Motil Cytoskeleton* 44: 209–224, 1999.
7. **Fassett JT, Xu X, Hu X, Zhu G, French J, Chen Y, Bache RJ.** Adenosine regulation of microtubule dynamics in cardiac hypertrophy. *Am J Physiol Heart Circ Physiol* 297: H523–H532, 2009.
8. **Foretz M, Ancellin N, Andreelli F, Saintillan Y, Grondin P, Kahn A, Thorens B, Vaulont S, Viollet B.** Short-term overexpression of a constitutively active form of AMP-activated protein kinase in the liver leads to mild hypoglycemia and fatty liver. *Diabetes* 54: 1331–1339, 2005.
9. **Frederick RL, Shaw JM.** Moving mitochondria: establishing distribution of an essential organelle. *Traffic* 8: 1668–1675, 2007.
10. **Gwinn DM, Shackelford DB, Egan DF, Mihaylova MM, Mery A, Vasquez DS, Turk BE, Shaw RJ.** AMPK phosphorylation of raptor mediates a metabolic checkpoint. *Mol Cell* 30: 214–226, 2008.
11. **Hu P, Zhang D, Swenson L, Chakrabarti G, Abel ED, Litwin SE.** Minimally invasive aortic banding in mice: effects of altered cardiomyocyte insulin signaling during pressure overload. *Am J Physiol Heart Circ Physiol* 285: H1261–H1269, 2003.
12. **Illenberger S, Drewes G, Trinczek B, Biernat J, Meyer HE, Olmsted JB, Mandelkow EM, Mandelkow E.** Phosphorylation of microtubule-associated proteins MAP2 and MAP4 by the protein kinase p110mark.

Downloaded from http://ajpheart.physiology.org/ at Univ of Minnesota on July 5, 2013

Phosphorylation sites and regulation of microtubule dynamics. *J Biol Chem* 271: 10834–10843, 1996.

13. **Inoki K, Zhu T, Guan KL.** TSC2 mediates cellular energy response to control cell growth and survival. *Cell* 115: 577–590, 2003.
14. **Iribe G, Ward CW, Camelliti P, Bollensdorff C, Mason F, Burton RA, Garny A, Morphew MK, Hoenger A, Lederer WJ, Kohl P.** Axial stretch of rat single ventricular cardiomyocytes causes an acute and transient increase in Ca^{2+} spark rate. *Circ Res* 104: 787–795, 2009.
15. **Koide M, Hamawaki M, Narishige T, Sato H, Nemoto S, DeFreyte G, Zile MR, Cooper G, Carabello BA.** Microtubule depolymerization normalizes in vivo myocardial contractile function in dogs with pressure-overload left ventricular hypertrophy. *Circulation* 102: 1045–1052, 2000.
16. **Lauf U, Giepmans BN, Lopez P, Braconnot S, Chen SC, Falk MM.** Dynamic trafficking and delivery of connexons to the plasma membrane and accretion to gap junctions in living cells. *Proc Natl Acad Sci USA* 99: 10446–10451, 2002.
17. **Mandelkow EM, Thies E, Trinczek B, Biernat J, Mandelkow E.** MARK/PAR1 kinase is a regulator of microtubule-dependent transport in axons. *J Cell Biol* 167: 99–110, 2004.
18. **Marx A, Nugoor C, Panneerselvam S, Mandelkow E.** Structure and function of polarity-inducing kinase family MARK/Par-1 within the branch of AMPK/Snf1-related kinases. *FASEB J* 24: 1637–1648, 2010.
19. **Matsushima K, Tokuraku K, Hasan MR, Kotani S.** Microtubule-associated protein 4 binds to actin filaments and modulates their properties. *J Biochem* 151: 99–108, 2012.
20. **Nakano A, Kato H, Watanabe T, Min KD, Yamazaki S, Asano Y, Seguchi O, Higo S, Shintani Y, Asanuma H, Asakura M, Minamino T, Kaibuchi K, Mochizuki N, Kitakaze M, Takashima S.** AMPK controls the speed of microtubule polymerization and directional cell migration through CLIP-170 phosphorylation. *Nat Cell Biol* 12: 583–590.
21. **Nakano A, Kato H, Watanabe T, Min KD, Yamazaki S, Asano Y, Seguchi O, Higo S, Shintani Y, Asanuma H, Asakura M, Minamino T, Kaibuchi K, Mochizuki N, Kitakaze M, Takashima S.** AMPK controls the speed of microtubule polymerization and directional cell migration through CLIP-170 phosphorylation. *Nat Cell Biol* 12: 583–590, 2010.
22. **Perhonen M, Sharp WW, Russell B.** Microtubules are needed for dispersal of alpha-myosin heavy chain mRNA in rat neonatal cardiac myocytes. *J Mol Cell Cardiol* 30: 1713–1722, 1998.
23. **Prosser BL, Ward CW, Lederer WJ.** X-ROS signaling: rapid mechano-chemo transduction in heart. *Science* 333: 1440–1445, 2011.
24. **Sato H, Nagai T, Kuppuswamy D, Narishige T, Koide M, Menick DR, Cooper G.** Microtubule stabilization in pressure overload cardiac hypertrophy. *J Cell Biol* 139: 963–973, 1997.
25. **Scholz D, Baicu CF, Tuxworth WJ, Xu L, Kasiganesan H, Menick DR, Cooper G.** Microtubule-dependent distribution of mRNA in adult cardiocytes. *Am J Physiol Heart Circ Physiol* 294: H1135–H1144, 2008.
26. **Scholz D, McDermott P, Garnovskaya M, Gallien TN, Huettelmaier S, DeRienzo C, Cooper G.** Microtubule-associated protein-4 (MAP-4) inhibits microtubule-dependent distribution of mRNA in isolated neonatal cardiocytes. *Cardiovasc Res* 71: 506–516, 2006.
27. **Scholz D, McDermott P, Garnovskaya M, Gallien TN, Huettelmaier S, DeRienzo C, and Cooper GT.** Microtubule-associated protein-4 (MAP-4) inhibits microtubule-dependent distribution of mRNA in isolated neonatal cardiocytes. *Cardiovasc Res* 71: 506–516, 2006.
28. **Shaw RJ.** LKB1 and AMP-activated protein kinase control of mTOR signalling and growth. *Acta Physiol (Oxf)* 196: 65–80, 2009.
29. **Shaw RM, Fay AJ, Puthenveedu MA, von Zastrow M, Jan YN, Jan LY.** Microtubule plus-end-tracking proteins target gap junctions directly from the cell interior to adherens junctions. *Cell* 128: 547–560, 2007.
30. **Shende P, Plaisance I, Morandi C, Pellieux C, Berthonneche C, Zorzato F, Krishnan J, Lerch R, Hall MN, Ruegg MA, Pedrazzini T, Brink M.** Cardiac raptor ablation impairs adaptive hypertrophy, alters metabolic gene expression, and causes heart failure in mice. *Circulation* 123: 1073–1082, 2011.
31. **Shioi T, McMullen JR, Tarnavski O, Converso K, Sherwood MC, Manning WJ, Izumo S.** Rapamycin attenuates load-induced cardiac hypertrophy in mice. *Circulation* 107: 1664–1670, 2003.
32. **Smyth JW, Hong TT, Gao D, Vogan JM, Jensen BC, Fong TS, Simpson PC, Stainier DY, Chi NC, Shaw RM.** Limited forward trafficking of connexin 43 reduces cell-cell coupling in stressed human and mouse myocardium. *J Clin Invest* 120: 266–279, 2010.
33. **Song X, Kusakari Y, Xiao CY, Kinsella SD, Rosenberg MA, Scherrer-Crosbie M, Hara K, Rosenzweig A, Matsui T.** mTOR attenuates the inflammatory response in cardiomyocytes and prevents cardiac dysfunction in pathological hypertrophy. *Am J Physiol Cell Physiol* 299: C1256–C1266, 2010.
34. **Stapleton D, Mitchelhill KI, Gao G, Widmer J, Michell BJ, Teh T, House CM, Fernandez CS, Cox T, Witters LA, Kemp BE.** Mammalian AMP-activated protein kinase subfamily. *J Biol Chem* 271: 611–614, 1996.
35. **Tagawa H, Koide M, Sato H, Zile MR, Carabello BA, Cooper G.** Cytoskeletal role in the transition from compensated to decompensated hypertrophy during adult canine left ventricular pressure overloading. *Circ Res* 82: 751–761, 1998.
36. **Tagawa H, Wang N, Narishige T, Ingber DE, Zile MR, Cooper G.** Cytoskeletal mechanics in pressure-overload cardiac hypertrophy. *Circ Res* 80: 281–289, 1997.
37. **Takahashi M, Shiraishi H, Ishibashi Y, Blade KL, McDermott PJ, Menick DR, Kuppuswamy D, Cooper G.** Phenotypic consequences of beta1-tubulin expression and MAP4 decoration of microtubules in adult cardiocytes. *Am J Physiol Heart Circ Physiol* 285: H2072–H2083, 2003.
38. **Terasaki M, Chen LB, Fujiwara K.** Microtubules and the endoplasmic reticulum are highly interdependent structures. *J Cell Biol* 103: 1557–1568, 1986.
39. **Thornton C, Bright NJ, Sastre M, Muckett PJ, Carling D.** AMP-activated protein kinase (AMPK) is a tau kinase, activated in response to amyloid beta-peptide exposure. *Biochem J* 434: 503–512, 2011.
40. **Thyberg J, Moskalewski S.** Role of microtubules in the organization of the Golgi complex. *Exp Cell Res* 246: 263–279, 1999.
41. **Tsutsui H, Ishihara K, Cooper G.** Cytoskeletal role in the contractile dysfunction of hypertrophied myocardium. *Science* 260: 682–687, 1993.
42. **Welte MA.** Bidirectional transport along microtubules. *Curr Biol* 14: R525–R537, 2004.
43. **Yaffe MP, Harata D, Verde F, Eddison M, Toda T, Nurse P.** Microtubules mediate mitochondrial distribution in fission yeast. *Proc Natl Acad Sci USA* 93: 11664–11668, 1996.
44. **Yi M, Weaver D, Hajnoczky G.** Control of mitochondrial motility and distribution by the calcium signal: a homeostatic circuit. *J Cell Biol* 167: 661–672, 2004.
45. **Zhang D, Contu R, Latronico MV, Zhang J, Rizzi R, Catalucci D, Miyamoto S, Huang K, Ceci M, Gu Y, Dalton ND, Peterson KL, Guan KL, Brown JH, Chen J, Sonenberg N, Condorelli G.** MTORC1 regulates cardiac function and myocyte survival through 4E-BP1 inhibition in mice. *J Clin Invest* 120: 2805–2816, 2010.
46. **Zhang P, Hu X, Xu X, Fassett J, Zhu G, Viollet B, Xu W, Wiczer B, Bernlohr DA, Bache RJ, Chen Y.** AMP activated protein kinase-alpha2 deficiency exacerbates pressure-overload-induced left ventricular hypertrophy and dysfunction in mice. *Hypertension* 52: 918–924, 2008.

Downloaded from http://ajpheart.physiology.org/ at Univ of Minnesota on July 5, 2013
